# A multi-enzyme cascade for efficient production of d-*p-*hydroxyphenylglycine from l-tyrosine

**DOI:** 10.1186/s40643-021-00394-2

**Published:** 2021-05-21

**Authors:** Xu Tan, Sheng Zhang, Wei Song, Jia Liu, Cong Gao, Xiulai Chen, Liming Liu, Jing Wu

**Affiliations:** 1 School of Pharmaceutical Science, Jiangnan University, 1800 Lihu Road, Wuxi, 214122 China; 2 State Key Laboratory of Food Science and Technology, Jiangnan University, Wuxi, 214122 China; 3 Zhejiang Tianrui Chemical Co., Ltd, Quzhou, 324400 China

**Keywords:** d-*p*-hydroxyphenylglycine, *meso*-diaminopimelate dehydrogenase, Hydride transfer distance, l-tyrosine, Protein engineering

## Abstract

In this study, a four-enzyme cascade pathway was developed and reconstructed in vivo for the production of d-*p*-hydroxyphenylglycine (D-HPG), a valuable intermediate used to produce β-lactam antibiotics and in fine-chemical synthesis, from l-tyrosine. In this pathway, catalytic conversion of the intermediate 4-hydroxyphenylglyoxalate by *meso*-diaminopimelate dehydrogenase from *Corynebacterium glutamicum* (*Cg*DAPDH) was identified as the rate-limiting step, followed by application of a mechanism-guided “conformation rotation” strategy to decrease the hydride-transfer distance d_(C6HDAP−C4NNADP)_ and increase *Cg*DAPDH activity. Introduction of the best variant generated by protein engineering (*Cg*DAPDH^BC621/D120S/W144S/I169P^ with 5.32 ± 0.85 U·mg^−1^ specific activity) into the designed pathway resulted in a D-HPG titer of 42.69 g/L from 50-g/L l-tyrosine in 24 h, with 92.5% conversion, 71.5% isolated yield, and > 99% enantiomeric excess in a 3-L fermenter. This four-enzyme cascade provides an efficient enzymatic approach for the industrial production of D-HPG from cheap amino acids.

**Supplementary Information:**

The online version contains supplementary material available at 10.1186/s40643-021-00394-2.

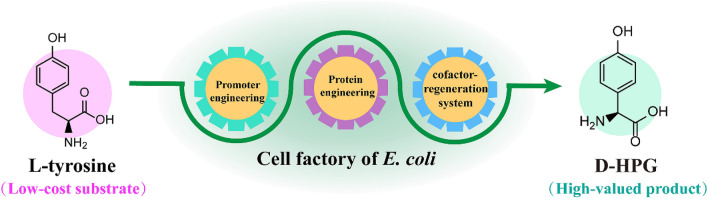

## Introduction

d-*p*-Hydroxyphenylglycine (D-HPG) is an important intermediate that is widely used in the pharmaceutical and fine-chemical industries for the production of β-lactam antibiotics (e.g., amoxicillin) and aromatic aldehydes (e.g., 4-hydroxybenzaldehyde) (Tripathi et al. 2000; Zhang and Cai 2014; Zhang et al. 2010). Currently, D-HPG is in high demand with a total annual market volume of ~ 10,000 tons (Li et al. 2019); therefore, the development of a practical method for the efficient production of D-HPG is required to meet this increasing market demand.

Two main strategies have been developed for D-HPG production: chemical synthesis and enzymatic synthesis. Approaches for the chemical synthesis of D-HPG mainly include chiral separation and esterification coupled with hydrolysis (Pollegioni et al. 2020). Using benzenesulfonic acid as a chiral agent, D-HPG can be separated from dl-*p*-hydroxyphenylglycine (DL-HPG) (Zhao and Xu 2015); however, the optical purity of D-HPG obtained by chiral separation is unsatisfactory, with further purification required. Esterification-coupled hydrolysis has emerged as a suitable technique to facilitate the industrial production of D-HPG. In this process, DL-HPG is first esterified with thionyl chloride to generate DL-HPG methyl ester, followed by hydrolysis to generate D-HPG (Zhang et al. 2011). Alternatively, enzyme catalysis provides a promising and efficient approach for synthesizing chiral chemicals (Wiltschi et al. 2020; Wu et al. 2021; Xue et al. 2018), such as (*R*)-β-tyrosine, (*R*)-phenyllactic acid, and l-homophenylalanine, which are commonly used in the synthesis of pharmaceuticals, cosmetics, and fine chemicals (Song et al. 2018; Wang et al. 2020). Accordingly, an enzymatic catalysis strategy involving dual-enzyme synthesis was developed, with this process comprising ring opening of dl-hydroxyphenylhydantoin (DL-HPH) and hydrolysis, catalyzed by d-hydantoinase (Hase; EC 3.5.2.2) and *N*-carbamoyl-d-amino-acid hydrolase (Case; EC 3.5.1.77), respectively (Aranaz et al. 2015; Diez et al. 2015; Liu et al. 2019). A previous study demonstrated this technique in *Escherichia coli* co-expressing Hase and Case, reporting production of 140 mM D-HPG from 140 mM DL-HPH after 32 h with a 100% yield and 0.73 g·L^−1^ h^−1^ productivity (Liu et al. 2019). Unfortunately, the low productivity and high cost of DL-HPH significantly limit industrial application of this process. Therefore, development of an efficient D-HPG-synthesis method remains challenging.

**Fig. 1:**
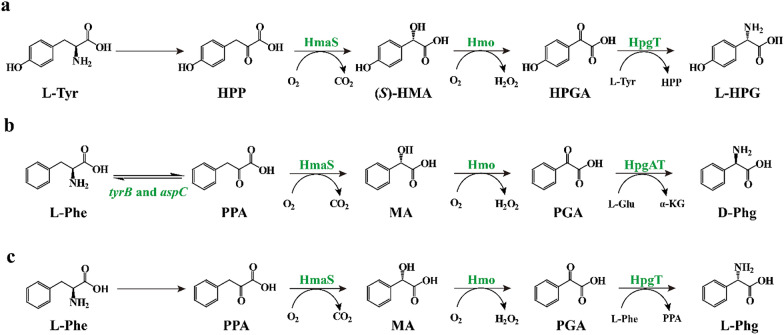
Biosynthetic pathway of phenylglycine analogues. **a** Biocatalysis of l-tyrosine to L-HPG. **b** Biocatalysis of L-Phe to D-Phg. **c** Biocatalysis of L-Phe to L-Phg

A biosynthetic pathway of vancomycin group antibiotics was characterized and modified to produce three phenylglycine analogues, including l-*p*-hydroxyphenylglycine (L-HPG), d-phenylglycine (D-Phg), and l-phenylglycine (L-Phg). As illustrated in Fig. 1a, the biosynthetic pathway with l-tyrosine as a substrate, and 4-hydroxymandelate synthase (HmaS; EC 1.13.11.46), 4-hydroxymandelate oxidase (Hmo; EC 1.1.3.46), and (*S*)-3,5-dihydroxyphenylglycine transaminase (HpgT; EC 2.6.1.103) as catalysts enabled the synthesis of L-HPG, which is a crucial component of certain peptidic natural products (Choroba et al. 2000; Hubbard et al. 2000). On this basis, an artificial D-Phg biosynthesis pathway harboring HmaS, Hmo, and d-4-hydroxyphenylglycine transaminase (HpgAT; EC 2.6.1.72) was created and introduced into an l-phenylalanine-producing *E. coli* strain (Fig. 1b) (Muller et al. 2006). After deleting the genes encoding the main aminotransferases for byproduct l-phenylalanine synthesis, *tyrB* and *aspC*, 102 ± 6 mg/g DCW D-Phg was generated from l-phenylalanine. Recently, Liu et al. reported a strategy by which HpgAT was replaced with HpgT using L-Phe as the amino donor to develop an artificial cascade route for L-Phg production (Fig. 1c) (Liu et al. 2014a, 2016). Further optimization of the expression of HmaS, Hmo, and HpgT, along with attenuation of L-Phe transamination resulted in up to 51.6 mg/g DCW of L-Phg. Although this pathway shows great potential to produce D-HPG, there are two obvious disadvantages that remain to be solved: (1) the oxidation process catalyzed by Hmo accumulates cytotoxic H_2_O_2_, which requires additional catalase for consumption of H_2_O_2_, thus complicating the reaction process; and (2) the transamination processes catalyzed by HpgT or HpgAT require an amino donor as the co-substrate, resulting in a large increase in the total reaction costs. Considering these limitations, it is urgent to develop suitable catalytic enzymes.

*meso*-Diaminopimelate dehydrogenase (DAPDH; EC 1.4.1.16) is an excellent enzyme for converting bulky aromatic α-keto acids to the corresponding d-amino acids (Ahmed et al. 2015; Gao et al. 2012, 2017). In recent decades, the catalytic activity toward bulky aromatic α-keto acids has been improved as a consequence of protein engineering (Akita et al. 2012; Gao et al. 2013; Liu et al. 2014b). For example, mutation of *Ureibacillus thermosphaericus* DAPDH (*Ut*DAPDH^D94A^) resulted in an 8.3-fold increase in enzyme activity toward various bulky α-keto acids such as phenylpyruvic acid (Hayashi et al. 2017). Correspondingly, the substrate scope of DAPDH could also be broadened. For example, DAPDH from *Clostridium tetani* E88 (*Ct*DAPDH) showed no detectable activity toward phenylpyruvic acid, whereas the activity of the variant strain with several introduced mutations (*Ct*DAPDH^Q154L/T173I/R199M/P248S/H249N/N276S^) toward phenylpyruvic acid increased to 0.11 U·mg^−1^ (Liu et al. 2015). Therefore, DAPDH could be employed to support the reductive amination of 4-hydroxyphenylglyoxalate (HPGA) to D-HPG.

In this study, a four-enzyme cascade pathway enabling the transformation of l-tyrosine to D-HPG was developed and the pathway was reconstructed in vivo. To increase the D-HPG titer, a mechanism-guided “conformation rotation” strategy was further developed to improve the catalytic activity of *Cg*DAPDH toward HPGA. Incorporating the optimal *Cg*DAPDH variant into the cascade pathway enabled the synthesis of 42.69 g/L D-HPG from 50 g/L l-tyrosine in 24 h with 92.5% conversion, 71.5% isolated yield, and > 99% enantiomeric excess (*ee*) in a 3-L scale fermenter.

## Methods

### Reagents

Commercial reagents, standards, and solvents were purchased from Sigma-Aldrich (Shanghai, China), Meryer Chemicals (Shanghai, China), Aladdin Reagents (Shanghai, China), J&K Chemicals (Beijing, China), and TCI Chemicals (Shanghai, China), and used without further purification.

### Strains and plasmids

The host strain *Escherichia coli* BL21 (DE3) was purchased from Invitrogen (Carlsbad, CA, U.S.A.), and was used for all molecular cloning and biotransformation experiments. Gene expression was achieved by cloning the desired gene(s) into a set of plasmids pET28a (+), pETDuet-1, and pACYCDuet-1 (Novagen, Darmstadt, Germany). Main plasmids and strains construction are listed in Table 1.

**Table 1:** Host strains and plasmids used in this study

Strain^a^	Recombinant plasmids^b^ in the strain
*E. coli* 01	pACYC-*Pm*L-AAD-*Samb*HmaS, pET-*Pa*MDH-*Cg*DAPDH^BC621^
*E. coli* 02	pET-_(Tac)_*Pa*MDH-*Cg*DAPDH^BC621^
*E. coli* 03	pET-_(T5)_*Pa*MDH-*Cg*DAPDH^BC621^
*E. coli* 04	pET-_(Tre)_*Pa*MDH-*Cg*DAPDH^BC621^
*E. coli* 05	pET-_(Trp)_*Pa*MDH-*Cg*DAPDH^BC621^
*E. coli* 06	pET-_(T7)_*Pa*MDH-*Cg*DAPDH^BC621^
*E. coli* 07	pACYC-*Pm*L-AAD-*Samb*HmaS, pET-_(Tac)_*Pa*MDH-*Cg*DAPDH^BC621/D120S/W144S/I169P^

L-AAD: l-amino acid deaminase; HmaS: 4-hydroxymandelate synthase; MDH: (*S*)-mandelate dehydrogenase; DAPDH: *meso*-diaminopimelate dehydrogenase

^a^The strains were constructed by transforming the corresponding recombinant plasmids into *E. coli* BL21 (DE3) express strains (New England Biolabs)

^b^The recombinant plasmids were constructed on pACYCDuet-1 or pETDuet-1 (Novagen)

### Homology modeling

The 3D homology modeling structures of catalytic domain of *Cg*DAPDH^BC621^ and its variants were constructed using the SWISS-MODEL online server (http://swissmodel.expasy.org/) with DAPDH from *Corynebacterium glutamicum* as template (*Cg*DAPDH, 99% identity, PDB ID: 5LOA). Quality assessment of the resulting models was performed using the Verify 3D and Procheck programs in SAVE V5.0 (http://servicesn.mbi.ucla.edu/SAVES/). Verify 3D determines the compatibility of a 3D atomic model with its own 1D amino acid sequence by assigning a structural class, and comparing the results to well-resolved structures (Bowie et al. 1991; Luthy et al. 1992). At least 80% of residues with a 3D-1D score ≥ 0.2 are qualified. The evaluation results showed that 98.12% of the residues have an average 3D-1D score ≥ 0.2, indicating that the quality of the model met the requirements (Additional file 1: Fig. S1). The Procheck program uses Ramachandran plots to reflect the stereochemical quality of a protein structure by analyzing the residue-by-residue geometry and overall structure geometry (Laskowski et al. 1993). A good-quality model would be expected to have over 90% amino acid residues in the most favored regions. Evaluation of Ramachandran plots showed that 93.9% of the residues were in the most favored regions, indicating that the quality of the model was acceptable (Additional file 1: Fig. S2). The Ramachandran plots also showed that only residue D218 was present in the unfavorable region. Further analysis showed that D218 is far away from the active center and does not affect the accuracy of the *Cg*DAPDH^BC621^ structural model. Model optimization was performed by 5-ns dynamics simulations using GROMACS with the GROMOS96 54a7 force field. The protein conformation of the last frame was extracted as the optimal *Cg*DAPDH^BC621^ model for subsequent molecular docking and molecular dynamics (MD) simulation analyses (Additional file 1: Fig. S3).

The 3D structures of D-HPG, HPGA, and NADP^+^ were downloaded from the PubChem Compound (https://www.ncbi.nlm.nih.gov/pccompound/). The analysis of the structures was performed by PyMOL 2.2 (by Schrodinger (SDGR) company). Docking simulations were performed using Autodock Vina and *Cg*DAPDH^BC621^ models.

### MD simulations

The MD simulations were performed using GROMACS with the GROMOS96 54a7 force field following the three main steps of energy minimization, system equilibration, and production protocols (Abraham et al. 2015). After the energy minimization, the systems were gently heated using six 50-ps steps, incrementing the temperature 50 K each step (0–300 K, 30 °C) under constant volume and periodic boundary conditions. Finally, 20-ns MD simulation under NVT ensemble was performed with an integration time step of 2 fs via use of the periodic boundary condition. All simulations were performed individually for both the complexes of *Cg*DAPDH^BC621^ and its mutants. The MD simulations results were analyzed in GROMACS. The difference of root‐mean‐square deviation (RMSD) between *Cg*DAPDH^BC621^ and *Cg*DAPDH^BC621/D120S/W144S/I169P^ were calculated in the last 15 ns when the values were balanced. The flexible region A identified by MD simulations comprised residues W144 to Y168, and the flexible region B comprised residues T42 to V61.

### HPLC analysis

Identification of products was accomplished by HPLC analysis based on the integration of monomer peaks using external commercial standards. Analysis of the concentration and *ee* of D-HPG was conducted using Agilent 1260 HPLC with Daicel Crownpak CR‐I (+) column (150 × 3 mm, 5 μm; Daicel Co., Japan) and pH 1.5 HClO_4_ a.q./acetonitrile (90/10, v/v) as the mobile phase. Flow: 0.2 mL/min, temperature: 25 °C, wavelength: 253 nm.

### Directed evolution experiments

#### Construction of CgDAPDH variants

The mutant library was constructed by whole-plasmid PCR using PrimeSTAR®HS (TaKaRa) and plasmid pET28a-*Cg*DAPDH^BC621^ as the template. The primers used for variants construction are listed in Additional file 1: Table S1. The resultant PCR products were digested with DpnI to eliminate the template plasmid. After elimination, 10 μL of digested products were transformed into *E. coli* BL21 (DE3) cells for the following screening or DNA sequencing (GENEWIZ, China).

#### Cultivation and expression of the mutants in 96-deep-well plates

The single colonies in culture dishes were randomly picked and cultured into 500 μL LB medium with 50-μg/mL kanamycin in 96-deepwell plates and shaken at 37 °C for 12 h. Then, they were 1:10 diluted into 500 μL fresh medium in new 96-deepwell plates (containing 2-g/L glucose and 4-g/L lactose). After shaking at 37 °C for 3 h (for cell growth), the temperature was decreased to 25 °C for 15 h (for protein expression). Then, the cells were harvested by centrifugation at 3600×*g* at 4 °C for 15 min. The cells were resuspended in 200 μL of the same buffer with 2-mg/mL lysozyme and 0.1% Triton X-100 and the mixture was incubated at 37 °C for 2 h with shaking. Finally, the crude extract was obtained by centrifugation at 3600 × *g* for 15 min at 4 °C.

#### High-throughput screening

After centrifugation, 50-μL supernatant was added into a new 96-well plate containing 500 μL Tris–HCl buffer (50 mM, pH 8.0), 10-mM HPGA, and 0.5-mM NADPH, and then incubated at 30 °C for 24 h. The same volume of a dye mixture containing 0.01 g/L of phenazine methosulfate (PMS) and 0.2 g/L of nitroblue tetrazolium (NBT) was then added to the reaction mixture. The mixture was analyzed for activity using a formazan-based method that NADPH reacts with NBT to produce formazan, which could be monitored at 590 nm using BioTek Synergy microplate reader, in the presence of PMS. The absorbance ratios was coupled to D-HPG titer and the absorbance at 590 nm for each candidate residue of the site-saturation libraries relative to those of *Cg*DAPDH^BC621^ were calculated. Only when the absorbance of ratio ≤ 0.8, the strain was sequenced and tested in shaking flasks.

### Fermentation medium and conditions

#### Shaking culture

A single colony of recombinant *E. coli* strain was cultivated overnight (10–12 h, 37 °C) in LB medium (10-g/L peptone, 5-g/L yeast extract, and 10-g/L NaCl; pH 7.0) with appropriate antibiotics (50-μg/mL kanamycin or 100-μg/mL ampicillin) and used as the inoculum (1%). The culture was then transferred into 50-mL Terrific Broth (TB) medium (24-g/L yeast extract, 12-g/L tryptone, 5-g/L glucose, 2.31-g/L KH_2_PO_4_, and 16.43-g/L K_2_HPO_4_; pH 7.0) containing appropriate antibiotics in a 500-mL flask. When the OD_600_ of the culture broth reached 0.6–0.8, isopropyl β-D-1-thiogalactopyranoside (IPTG) was added to a final concentration of 0.4 mM to induce gene expression. The cells were inducted at 25 °C for 15 h and collected by centrifugation (6000×*g*, 8 min). Then, the cell pellets were resuspended in an appropriate buffer to the desired density as resting cells for biotransformation.

#### Fermentor (3 L) culture

Additional larger fermentations were conducted in a 3-L fermentation system (INFORS HT Labfors, Switzerland) with an air flow rate of 1.5 vvm and a stirrer speed of 500 rpm. The pH was maintained at 7.0 by automatically feeding concentrated carbon and nitrogen resources (400-g/L glucose, 100-g/L yeast extract, and 25-g/L tryptone; start feeding after a steep rise in dissolved oxygen, 14 mL/h). Enzyme expression was induced at 25 °C with 0.4-mM IPTG (final concentration) at an optical density of 4 at 600 nm. Pre-cultures were grown in 500-mL flasks as described above. The cell pellets were collected for preparative biotransformation after 12-h induction.

### Enzyme purification

The recombinant *E. coli* strains containing *Pm*L-AAD, *Samb*HmaS, *Pa*MDH, *Cg*DAPDH, and *Cg*DAPDH variants were cultured in LB medium-containing kanamycin (50 μg/mL) at 37 °C and 200 rpm. When the culture’s optical density (OD_600_) reached 0.6–0.8, 0.4-mM IPTG (final concentration) was added to induce enzyme expression at 25 °C for an additional 15 h. The cells were harvested by centrifugation (6000×*g*, 10 min) at 4 °C, and resuspended in buffer A (25-mM Tris, 20-mM imidazole, 150-mM NaCl, pH 8.0; 10 mL/g of wet weight). The cell suspensions were lysed by sonication and the lysate containing l-amino acid deaminase (L-AAD) was treated with Tween 80 as a surfactant to dissolve L-AAD for 2 h, followed by centrifugation at 14,000×*g* for 30 min. The lysate containing HmaS, (*S*)-mandelate dehydrogenase (MDH), or DAPDH was directly centrifuged at 14,000×*g* for 30 min. The subsequent experiments were performed on an ÄKTA pure system (GE Healthcare) with a HisTrap HP column (5 mL, GE Healthcare). Protein concentration of purified enzyme was measured by detecting absorbance at 280 nm using a NanoDrop 2000c spectrophotometer (Thermo Scientific) and taking into account the calculated extinction coefficients with the ExPASy ProtParam Tool. The purity of the proteins was determined by gel filtration and SDS-PAGE. All purification operations were conducted at 4 °C when necessary.

### Activity assay

The activity of L-AAD was determined by measuring the initial rate of deamination of l-tyrosine by HPLC under the following conditions: 10-μM purified L-AAD and 10-mM l-tyrosine in 1-mL Tris–HCl buffer (50 mM, pH 8.0) at 30 °C for 5 min. The reaction was stopped with centrifugation at 12,000×*g* for 5 min, and the samples were analyzed by HPLC. One unit of activity was defined as the amount of enzyme required for deaminizing 1-μM l-tyrosine per minute.

The activity of HmaS was determined by measuring the initial rate of oxidation of 4-hydroxyphenylpyruvate (HPP) by HPLC under the following conditions: 10-μM purified HmaS, 10-mM HPP, and 0.5-mM CoSO_4_ in 1-mL Tris–HCl buffer (50 mM, pH 8.0) at 30 °C for 5 min. The reaction was stopped with centrifugation at 12,000×*g* for 5 min, and samples were analyzed by HPLC. One unit of activity was defined as the amount of enzyme required for producing 1-μM (*S*)-4-hydroxymandelate (HMA) per minute.

The activity of MDH was determined by measuring the initial rate of oxidation of (*S*)-HMA by HPLC under the following conditions: 10-μM purified MDH, 10-mM (*S*)-HMA, and 0.5-mM NADP^+^ in 1-mL Tris–HCl buffer (50 mM, pH 8.0) at 30 °C for 5 min. The reaction was stopped with centrifugation at 12,000×*g* for 5 min, and samples were analyzed by HPLC. One unit of activity was defined as the amount of enzyme required for producing 1-μM HPGA per minute.

The activity of DAPDH was determined based on the change of NADPH absorbance at 340 nm under the following conditions: 10-μM purified DAPDH, 10-mM HPGA, 0.5-mM NADPH, and 20-mM NH_4_Cl in 1-mL Tris–HCl buffer (50 mM, pH 8.0) at 30 °C for 5 min. One unit of activity was defined as the amount of enzyme required for oxidizing 1-μM NADPH per minute.

The protein concentration was determined by the Bradford protocol, using bovine serum albumin as the standard. All experiments were conducted in triplicate.

### Kinetic assay

The kinetic parameters of enzymes were determined by measuring the initial rates of product formation at different concentrations of substrate (1–20 mM) for 5 min. Other assay conditions were the same as those described above for the corresponding activity assay. The samples were withdrawn, extracted, and analyzed by HPLC. The *K*_*m*_ and *k*_*cat*_ values were calculated by nonlinear regression according to the Michaelis–Menten equation using Origin software.

### Biotransformation procedures in a 3-L bioreactor

The conversion experiments were carried out in a 3-L bioreactor with 800-mL working volume. Recombinant *E. coli* 07 were used as whole-cell biocatalyst (20 g/L) to convert 50-g/L L-tyrosine to D-HPG. The reaction was conducted in 20-mM Tris–HCl buffer (pH 8.5, 0.5-mM CoSO_4_, 0.7-mM NADP^+^, and 50-g/L NH_4_Cl) at 500 rpm and 30 °C for 24 h. At the end of the reaction, 100 μL of supernatant was separated after centrifugation (12,000×*g*, 10 min), diluted with 900 μL mobile phase. The resulting solution was filtered through 0.22-μm membrane filters and analyzed by HPLC for quantifying the products under the conditions stated below.

## Results

### Cascade design and in vitro reconstruction of the D-HPG-biosynthesis pathway

**Fig. 2:**
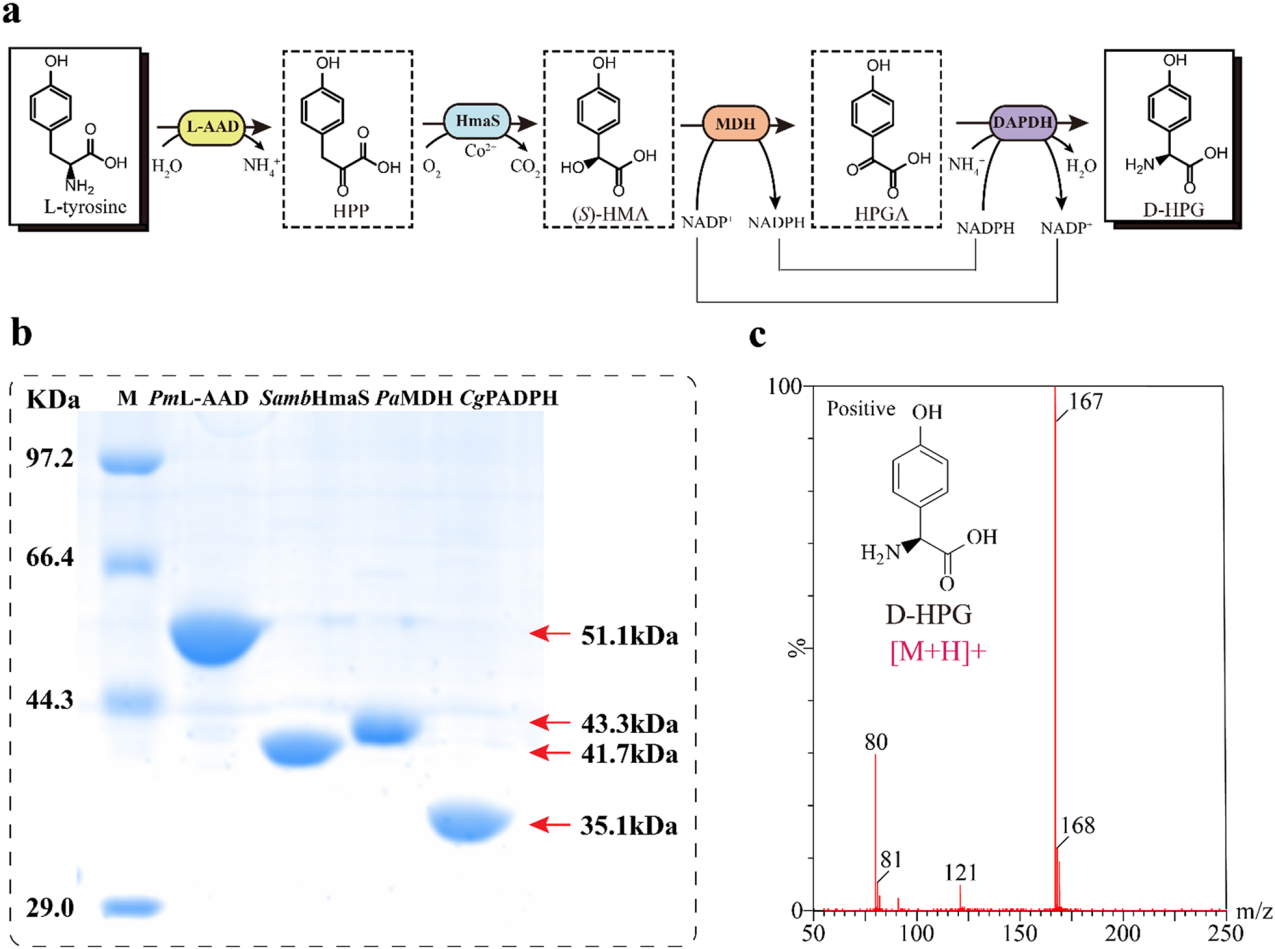
Cascade design and in vitro reconstruction of D-HPG biosynthesis pathway. **a** Schematic representation of D-HPG biosynthesis from L-tyrosine through a four-enzyme cascade. **b** Cascade pathway protein characterization. **c** Analysis of the in vitro reconstructed system with LC–MS. The four-enzyme system was supplemented with 10-mM L-tyrosine, 0.5-mM NADP^+^, 20-mM NH_4_Cl, and 0.5-mM CoSO_4_, and each of the four purified enzymes with 10 μM

Comparison of the structures of l-tyrosine and D-HPG showed that the l-tyrosine side chain contained one more carbon than the D-HPG side chain, and that the CH_2_ subunit at the α position of the l-tyrosine side chain cannot be removed by natural enzymes. Therefore, a sequential cascade was designed to synthesize D-HPG from l-tyrosine (Fig. 2a): first, l-tyrosine was deaminized to HPP by an L-AAD (EC 1.4.3.2), followed by HPP conversion to (*S*)-HMA via oxidative decarboxylation by HmaS. (*S*)-HMA was subsequently oxidized to HPGA by (*S*)-MDH (EC 1.1.99.31) and finally asymmetrically reduced to D-HPG by DAPDH (Fig. 2a).

To reconstruct this cascade pathway in vitro, a total of 20 different L-AAD, HmaS, MDH, and DAPDH enzymes from the BRENDA database were evaluated. An L-AAD from *Proteus mirabilis* (*Pm*L-AAD), HmaS from *Streptomyces ambofaciens* (*Samb*HmaS), MDH from *Pseudomonas aeruginosa* (*Pa*MDH), and a highly stereoselective DAPDH variant from *Corynebacterium glutamicum* (*Cg*DAPDH^BC621^ containing five mutations: R196M/T170I/H245N/Q151L/D155G) were subsequently selected according to their specific enzyme activities (Vedha et al. 2006) (Additional file 1: Tables S2–S5). The selected genes were then amplified, overexpressed, and purified (Fig. 2b). To confirm the feasibility of in vitro reconstruction, the four enzymes were employed at a molar ratio of 1:1:1:1 with 5-mM l-tyrosine. After a 4-h reaction, the formation of 0.85-mM D-HPG was confirmed as the final product by NMR and MS analysis (Fig. 2c and Additional file 1: Fig. S4), demonstrating the efficacy of the designed cascade using *Pm*L-AAD, *Samb*HmaS, *Pa*MDH, and *Cg*DAPDH^BC621^ for converting l-tyrosine to D-HPG.

### In vivo construction of the D-HPG biosynthesis pathway

**Fig. 3:**
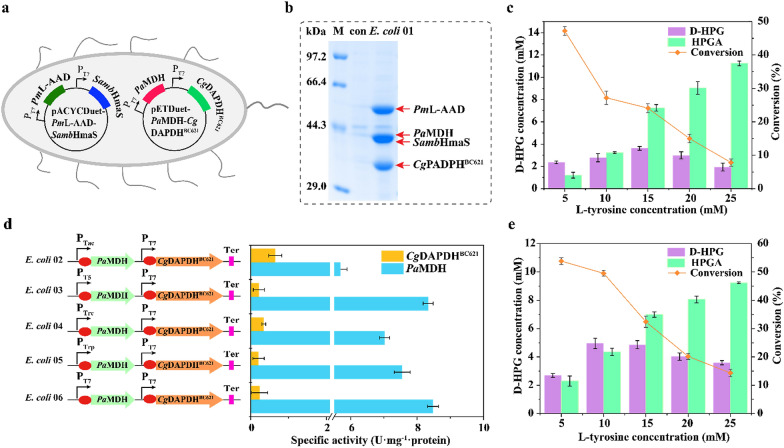
In vivo construction and optimization of multi-step cascade reactions. **a** Strain *E. coli* 01 containing double plasmids to express *Pm*L-AAD, *Samb*HmaS, *Pa*MDH, and *Cg*DAPDH^BC621^. **b** SDS-PAGE analysis of strain *E. coli* 01 from cell-free extracts. M, Maker; con, *E. coli* BL21 without overexpressing any enzymes. **c** Effect of substrate loading on D-HPG production by strain *E. coli* 01. **d** The specific activity of *Pa*MDH and *Cg*DAPDH^BC621^ in recombinant strains with different promoter sequences. **e** Effect of substrate loading on D-HPG production by strain *E. coli* 02. The reactions were supplemented with varying concentrations of L-tyrosine from 5 to 25 mM and 20-g/L wet cell added at 30 °C. The conversion and titer were obtained after completion of the reactions and determined by HPLC analysis. The values are averages of three experiments

To reconstruct this pathway in vivo, the genes encoding *Pm*L-AAD, *Samb*HmaS, *Pa*MDH, and *Cg*DAPDH^BC621^ were inserted into the pACYCDuet-1 and pETDuet-1 plasmids, which were then transformed into *E. coli* BL21 (DE3), resulting in strain *E. coli* 01 (Fig. 3a). Following confirmation of enzyme expression (Fig. 3b), the performance of *E. coli* 01 (20-g/L wet cells) was determined at 30 °C, revealing that the D-HPG titer increased from 2.35 ± 0.5 mM to 3.62 ± 0.9 mM along with increasing l-tyrosine concentration (5–15 mM) (Fig. 3c); however, at l-tyrosine concentrations > 15 mM, the D-HPG titer did not increase. Specifically, the molar conversion of D-HPG decreased from 47.2 ± 0.02% to 14.5 ± 0.2% along with an increase in the l-tyrosine concentration from 5 to 25 mM. This decrease was due to the accumulation of HPGA, a cascade intermediate, from 1.21 ± 0.2 mM to 11.25 ± 0.8 mM in the conversion broth (Fig. 3c). Furthermore, determination of the properties of *Pm*L-AAD, *Samb*HmaS, *Pa*MDH, and *Cg*DAPDH^BC621^ in *E. coli* 01 showed that *Pa*MDH exhibited high specific activity of 8.29 U·mg^−1^·protein, whereas *Cg*DAPDH^BC621^ continued to show low specific activity of 0.37 U·mg^−1^·protein, resulting in a *Pa*MDH: *Cg*DAPDH^BC621^ ratio of 22: 1 (Table 2). This result indicated that the imbalanced catalytic activities of the four enzymes promoted the accumulation of the intermediate HPGA, which prevented the continuous conversion of l-tyrosine to D-HPG.

**Table 2:** Kinetic constants of *Pm*L-AAD, *Samb*HmaS, *Pa*MDH, *Cg*DAPDH^BC621^ in *E. coli* 01

Enzyme	Specific activity (U·mg^−1^·protein)	*K*_*m*_ (mM)	*k*_*cat*_ (min^−1^)	*k*_*cat*_* /K*_*m*_ (mM^−1^·min^−1^)
*Pm*L-AAD	6.99 ± 0.37	2.94 ± 1.04	10.98 ± 0.52	3.73 ± 0.50
*Samb*HmaS	6.27 ± 0.69	0.72 ± 1.82	3.58 ± 1.27	4.97 ± 0.76
*Pa*MDH	8.29 ± 1.07	0.89 ± 0.91	7.36 ± 0.73	10.71 ± 0.22
*Cg*DAPDH^BC621^	0.37 ± 0.28	2.91 ± 0.30	0.25 ± 0.97	0.23 ± 0.02

L-AAD: l-amino acid deaminase; HmaS: 4-hydroxymandelate synthase; MDH: (*S*)-mandelate dehydrogenase; DAPDH: *meso*-diaminopimelate dehydrogenase

^a^The specific activity was determined with 10-μM purified enzymes and 10-mM corresponding substrate in 1-mL Tris–HCl buffer (50 mM, pH 8.0) at 30 °C for 15 min

^b^The *k*_*cat*_* /K*_*m*_ values was determined with 10-μM purified enzymes and 1–20-mM corresponding substrate in 1-mL Tris–HCl buffer (50 mM, pH 8.0) at 30 °C for 30 min

To control the expression levels of *Pa*MDH and *Cg*DAPDH^BC621^ in strain *E. coli* 01, four promoter sequences with lower activation strengths were selected to replace the T7 promoter in pETDuet-1, resulting in strains *E. coli* 02–06 (Fig. 3d, Fig. 3e and Additional file 1: Fig. S5). *E. coli* 02 showed significantly decreased specific activity for *Pa*MDH (5.69 ± 0.3 U·mg^−1^·protein) and increased specific activity of *Cg*DAPDH^BC621^ (0.65 ± 0.5 U·mg^−1^·protein) relative to that of *E. coli* 01, which was associated with the highest D-HPG titer (4.95 ± 0.5 mM) from 10-mM l-tyrosine but a low conversion rate of 49.5 ± 0.02%. To further increase the expression of *Cg*DAPDH^BC621^, the strategies of gene duplication and ribosome-binding sequence regulation were implemented; however, this did not increase the D-HPG titer, and HPGA accumulation remained high at 4.26 ± 0.04 mM. This was likely due to the insufficient *Cg*DAPDH^BC621^ activity for continuously transforming HPGA into D-HPG.

### Increasing *Cg*DAPDH activity by decreasing the d_(C6HDAP−C4NNADP)_ value

**Fig. 4:**
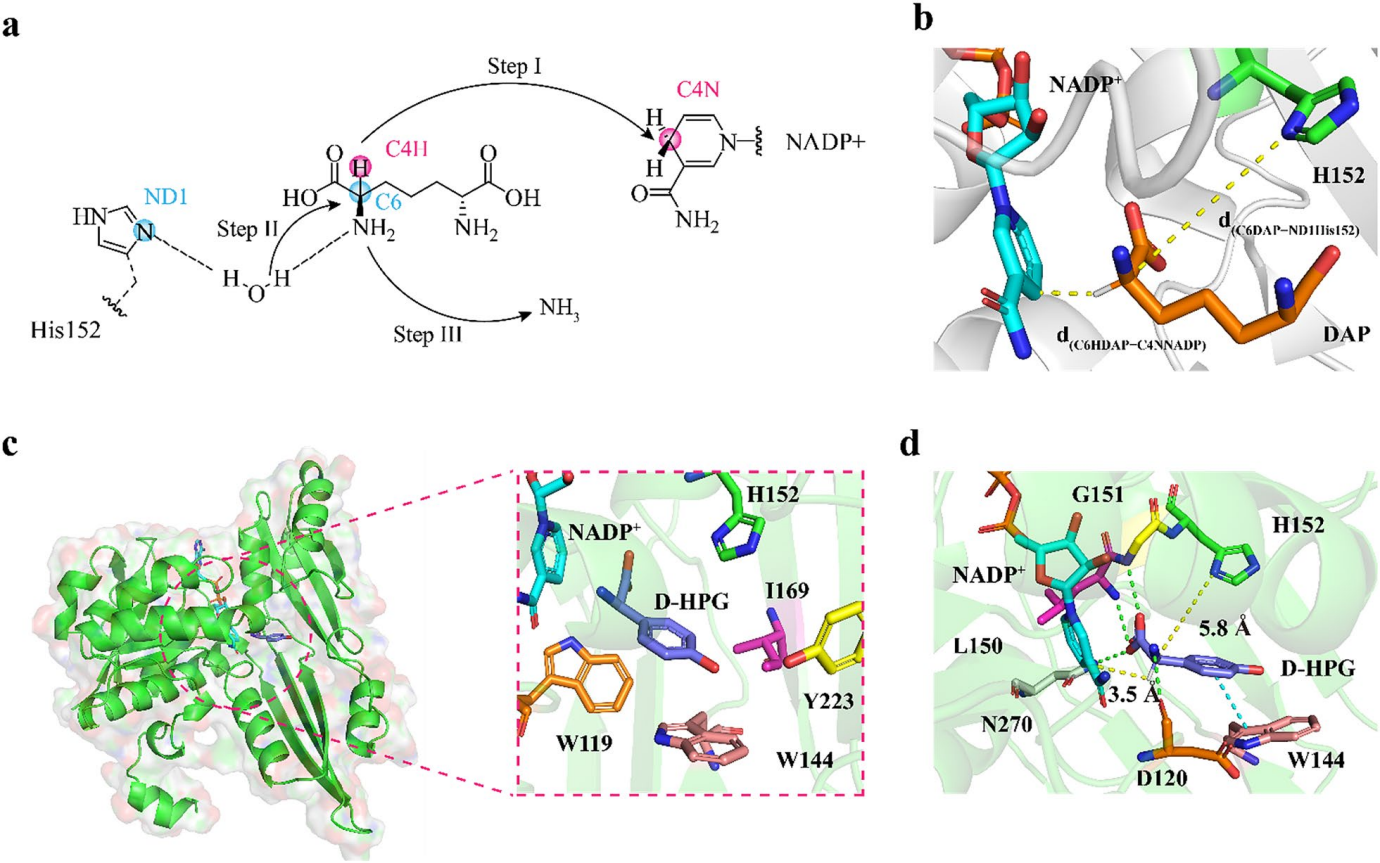
Putative mechanism of DAPDH catalysis and docking analysis of *Cg*DAPDH^BC621^-NADP^+^ with D-HPG. **a** A schematic view of the DAPDH reduction mechanism adapted to DAP. The hydride transfer from DAP (C4H) to NADP^+^ (C4N) is marked as pink spheres, the C6 of imino acid intermediate attacked by water and the ND1 of H152 are marked as blue spheres. **b** Two key distances in reductive amination process. The yellow dash lines denote d_(C6HDAP−C4NNADP)_ and d_(C6DAP−ND1His152)_, respectively. NADP^+^ cofactor is shown in cyan, DAP is shown in orange, and residue H152 is shown in green. **c** A detailed active site view of *Cg*DAPDH^BC621^-NADP^+^ with D-HPG. D-HPG is shown in purple, residue W119 is shown in orange, W144 is shown in light pink, I169 is shown in magenta, and Y223 is shown in yellow. **d** The interactions between D-HPG and *Cg*DAPDH^BC621^ and the two key distance values. The green dash lines denote hydrogen-bond interactions and the cyan dash line denote π–π stacking interaction. Residue D120 is shown in orange, L150 is shown in magenta, G151 is shown in yellow, and N270 is shown in white

As shown in Fig. 4a, the catalytic mechanism of DAPDH can be divided into three steps: (I) hydride (H^+^) transfer from the Cα of *meso*-diaminopimelate (DAP) to the C4N of the NADP^+^ nicotinamide ring, resulting in formation of an imino acid intermediate; (II) a water molecule attacks the imino acid intermediate to form a carbinolamine; and (III) *α*-keto acids and ammonia are released from the carbinolamine (Gao et al. 2019). According to this mechanism, two key distances were defined to represent the productive conformation (Fig. 4b): (1) the hydride-transfer distance [d_(C6HDAP−C4NNADP)_], which describes the distance between the hydrogen atom of DAP and the C4N atom of NADP^+^, representing the efficiency of hydride transfer in step I (2.3 Å < d_(C6HDAP−C4NNADP)_ < 2.7 Å); and (2) the distance related to the water-molecule attack in step II [d_(C6DAP−ND1His152)_], defined as the distance between the C6 atom of the substrate and the ND1 atom of H152 (6.0 Å < d_(C6DAP−ND1His152)_ < 6.8 Å) (Gao et al. 2019). It is speculated that the lower *Cg*DAPDH^BC621^ activity mainly originates from the inability of d_(C6HDAP−C4NNADP)_ or d_(C6DAP−ND1His152)_ to reach at the optimum range in the catalytic process of DAPDH.

A homology model of *Cg*DAPDH^BC621^ was reconstructed using SWISS-MODEL (https://swissmodel.expasy.org/) based on the crystal structure of *Cg*DAPDH (PDB ID: 5LOA) (Parmeggiani et al. 2016). Docking analysis using the *Cg*DAPDH^BC621^ structural model and D-HPG (Fig. 4c) revealed that W119 and W144 sterically hinder the benzene ring of D-HPG, and W144, H152, I169, and Y223 surround the phenolic hydroxyl group of D-HPG. Moreover, in the active site of *Cg*DAPDH^BC621^, four hydrogen bonds were identified between D-HPG with D120, L150, G151, and N270, as well as a π–π stacking interaction between D-HPG with the pyrrole ring of W144, anchoring D-HPG in the binding cavity. In this conformation, the d_(C6HDAP−C4NNADP)_ is 3.5 Å, which is higher than the suitable hydride-transfer distance (range 2.3–2.7 Å) (Fig. 4d). These findings indicated that D-HPG incompletely fit in the binding cavity, and the conformation of D-HPG is not beneficial to hydride transfer.

**Fig. 5:**
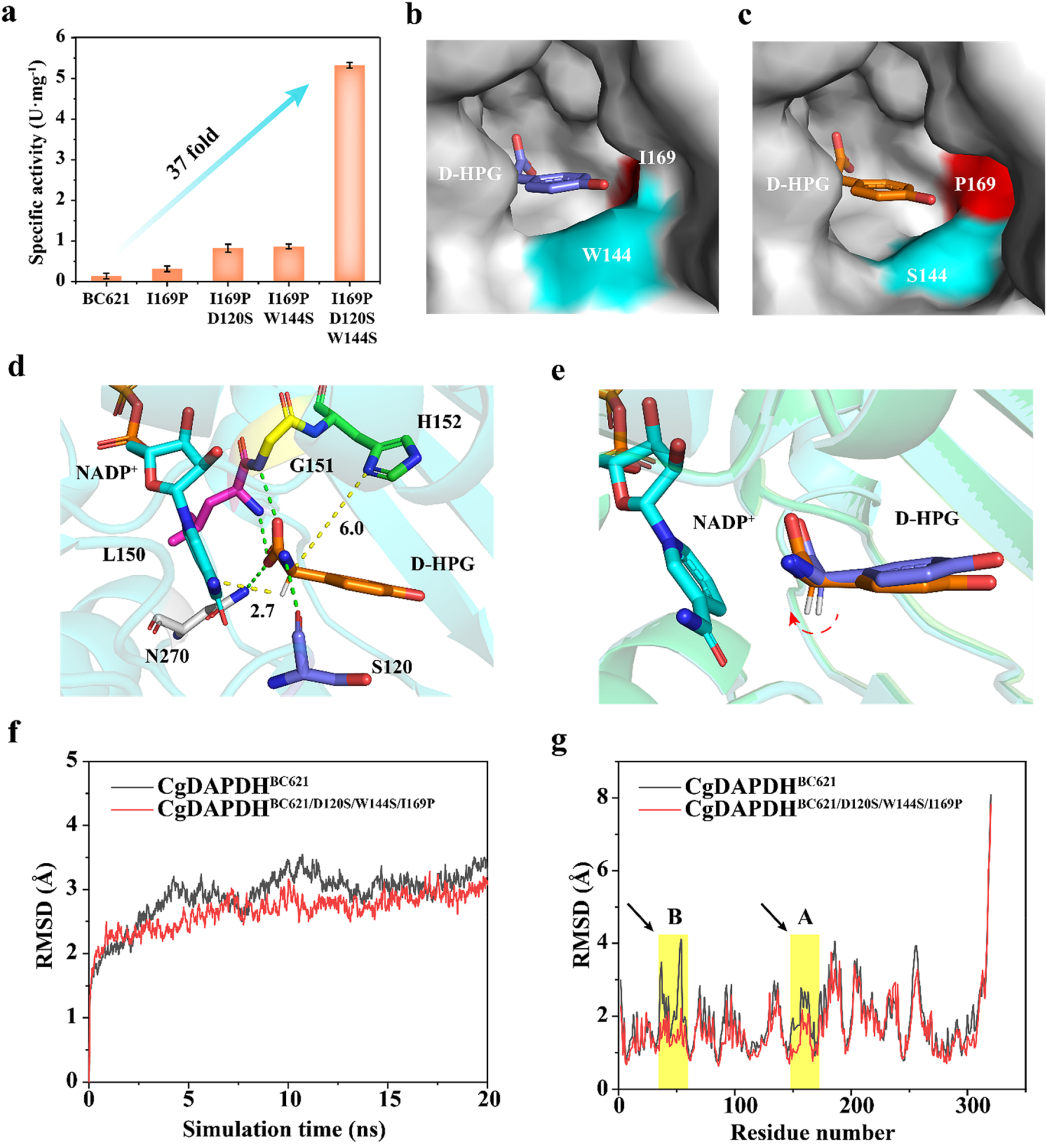
Improvement of the catalytic activity of *Cg*DAPDH^BC621^ toward HPGA by “conformational rotation” strategy. **a** Directed evolution of the parent *Cg*DAPDH^BC621^ for reductive amination of HPGA. **b** The binding pocket surface of *Cg*DAPDH^BC621^. D-HPG is shown in purple. **c** The binding pocket surface of *Cg*DAPDH^BC621/D120S/W144S/I169P^. D-HPG is shown in orange. **d** Docking of the D-HPG into the active site of *Cg*DAPDH^BC621/D120S/W144S/I169P^. D-HPG is shown in orange, NADP^+^ cofactor is shown in cyan, residue S120 is shown in purple, L150 is shown in magenta, G151 is shown in yellow, and H152 is shown in green. The hydrogen bond between D-HPG and residues S120, L150, G151, and N270 are shown in green dash lines, respectively. The yellow dash lines denote d_(C6HDAP−C4NNADP)_ and d_(C6DAP−ND1His152)_, respectively. **e** Superposition of the D-HPG conformation in parent *Cg*DAPDH^BC621^ and variant *Cg*DAPDH^BC621/D120S/W144S/I169P^. **f**–**g** RMSD values calculated from MD simulations of *Cg*DAPDH^BC621^ and *Cg*DAPDH^BC621/D120S/W144S/I169P^. The highlight represented the changes of the region with noticeable movements for *Cg*DAPDH^BC621^

Therefore, a “conformation rotation” strategy was employed to rotate the D-HPG conformation to decrease d_(C6HDAP−C4NNADP)_. Nine candidate residues (W119, D120, W144, L150, G151, H152, I169, N270, and Y223) were selected for NNK site-saturation mutagenesis. To efficiently screen potential positive variants, a formazan-based high-throughput method was developed that coupled the D-HPG titer with the absorbance at 590 nm. The lowest absorbance ratios for each mutated candidate residue relative to its variant in *Cg*DAPDH^BC621^ are shown in Additional file 1: Table S6. To rule out detection errors, only the variants with absorbance ratios ≤ 0.8 were selected and sequenced. Ultimately, four variants (*Cg*DAPDH^BC621/I169P^, *Cg*DAPDH^BC621/I169Y^, *Cg*DAPDH^BC621/D120S^, and *Cg*DAPDH^BC621/Y223C^) were identified, with *Cg*DAPDH^BC621/I169P^ showing a 1.3-fold increase in specific activity (0.32 ± 0.58-U·mg^−1^·protein) relative to that of *Cg*DAPDH^BC621^ (Additional file 1: Fig. S6). To further increase this activity, four recombinant variants were constructed: *Cg*DAPDH^BC621/D120S/I169P^, *Cg*DAPDH^BC621/I169P/Y223C^, *Cg*DAPDH^BC621/D120S/I169Y^, and *Cg*DAPDH^BC621/I169Y/Y223C^. Among these, *Cg*DAPDH^BC621/D120S/I169P^ presented a 4.3-fold increase in activity (0.74 ± 0.21-U·mg^−1^·protein) relative to that of *Cg*DAPDH^BC621^ (Additional file 1: Fig. S7). In addition, an iterative saturation variant library based on the *Cg*DAPDH^BC621/I169P^ variant was constructed, among which *Cg*DAPDH^BC621/W144S/I169P^ showed a 5.2-fold increase in activity relative to that of *Cg*DAPDH^BC621^ (Additional file 1: Fig. S8). Furthermore, combining the variants *Cg*DAPDH^BC621/D120S/I169P^ and *Cg*DAPDH^BC621/W144S/I169P^ to obtain *Cg*DAPDH^BC621/D120S/W144S/I169P^ resulted in a specific activity of 5.32 ± 0.85 U·mg^−1^·protein, which was 37-fold higher than that of *Cg*DAPDH^BC621^ (Fig. 5a).

Structural comparison of *Cg*DAPDH^BC621^ with *Cg*DAPDH^BC621/D120S/W144S/I169P^ (Fig. 5b and 5c) revealed that the D120S mutation decreases the length of the hydrogen bond with the amino group of D-HPG (from 3.0 to 2.9 Å), which in turn decreases the distance between L150 and the D-HPG carboxyl group (from 3.3 to 3.1 Å) (Additional file 1: Fig. S9), and the W144S mutation eliminated the π–π stacking interaction between W144 and D-HPG. Moreover, the I169P and W144S mutations increased the area for accommodating the phenol group of D-HPG, thereby providing D-HPG with a space for “conformational rotation” (Fig. 5e). These modifications decreased the d_(C6HDAP−C4NNADP)_ from 3.5 to 2.7 Å (Fig. 5d). Furthermore, MD simulations revealed that these modifications improved the stability of the conformation of variant *Cg*DAPDH^BC621/D120S/W144S/I169P^ (the RMSD decreased from 3.07 to 2.80 Å) (Fig. 5f), and decreased the flexibility of the residues in region A (W144 to Y168; RMSD decrease from 2.54 to 2.07 Å) and region B (T42 to V61; RMSD decrease from 3.52 to 1.91 Å) on the protein surface (Fig. 5g). These results indicated that inner interactions and the stability of variant *Cg*DAPDH^BC621/D120S/W144S/I169P^ might have been strengthened. As a result, the *K*_m_ value of *Cg*DAPDH^BC621/D120S/W144S/I169P^ (2.48 ± 0.28 mM) was 7.21-fold lower and the *k*_cat_ value (2.69 ± 0.30 min^−1^) was 13.16-fold higher than those of *Cg*DAPDH^BC621^ (20.37 ± 0.19 mM and 0.19 ± 0.37 min^−1^, respectively). This resulted in a 119-fold increase in the *k*_cat_/*K*_m_ value of *Cg*DAPDH^BC621/D120S/W144S/I169P^ (1.08 mM^−1^·min^−1^) relative to that of *Cg*DAPDH^BC621^ (Table 3).

**Table 3:** Kinetic constants of purified *Cg*DAPDH^BC621^ and its variants

Variants	Specific activity^a^ (U mg^−1^ protein)	*K*_*m*_ (mM)	*k*_*cat*_ (min^−1^)	*k*_*cat*_*/K*_*m*_^b^ (mM^−1^·min^−1^)
BC621	0.14 ± 0.18	20.37 ± 0.19	0.19 ± 0.37	0.009
I169P	0.32 ± 0.58	8.17 ± 0.89	1.48 ± 0.25	0.18
D120S/I169P	0.74 ± 0.21	7.83 ± 0.03	1.46 ± 0.70	0.19
W144S/I169P	0.87 ± 0.47	8.07 ± 0.68	1.35 ± 1.39	0.17
D120S/W144S/I169P	5.32 ± 0.85	2.48 ± 0.28	2.69 ± 0.30	1.08

DAPDH: *meso*-diaminopimelate dehydrogenase

^a^ The specific activity was determined with 10-μM purified *Cg*DAPDH^BC621^ or its variants and 10-mM HPGA in 1-mL Tris–HCl buffer (50-mM, pH 8.0, 20-mM NH_4_Cl) at 30 °C for 15 min

^b^ The *k*_*cat*_* /K*_*m*_ values was determined with 10-μM purified *Cg*DAPDH^BC621^ or its variants and 1–20-mM HPGA in 1-mL Tris–HCl buffer (50 mM, pH 8.0, 10-mM NH_4_Cl) at 30 °C for 30 min

### One-pot production of D-HPG at the 3-L scale

*Cg*DAPDH^BC621/D120S/W144S/I169P^ was used to replace *Cg*DAPDH^BC621^ in *E. coli* 02, resulting in *E. coli* 07 (Additional file 1: Fig. S10). After 24 h, the D-HPG titer increased to 9.03 ± 0.32 mM along with a 90.3 ± 0.03% molar conversion, which was a 0.82-fold increase relative to that of *E. coli* 02. Additionally, HPGA accumulation remained below 0.22 ± 0.06 mM, indicating that the catalytic activity of *Cg*DAPDH^BC621/D120S/W144S/I169P^ matched that of *Pa*MDH.

**Fig. 6:**
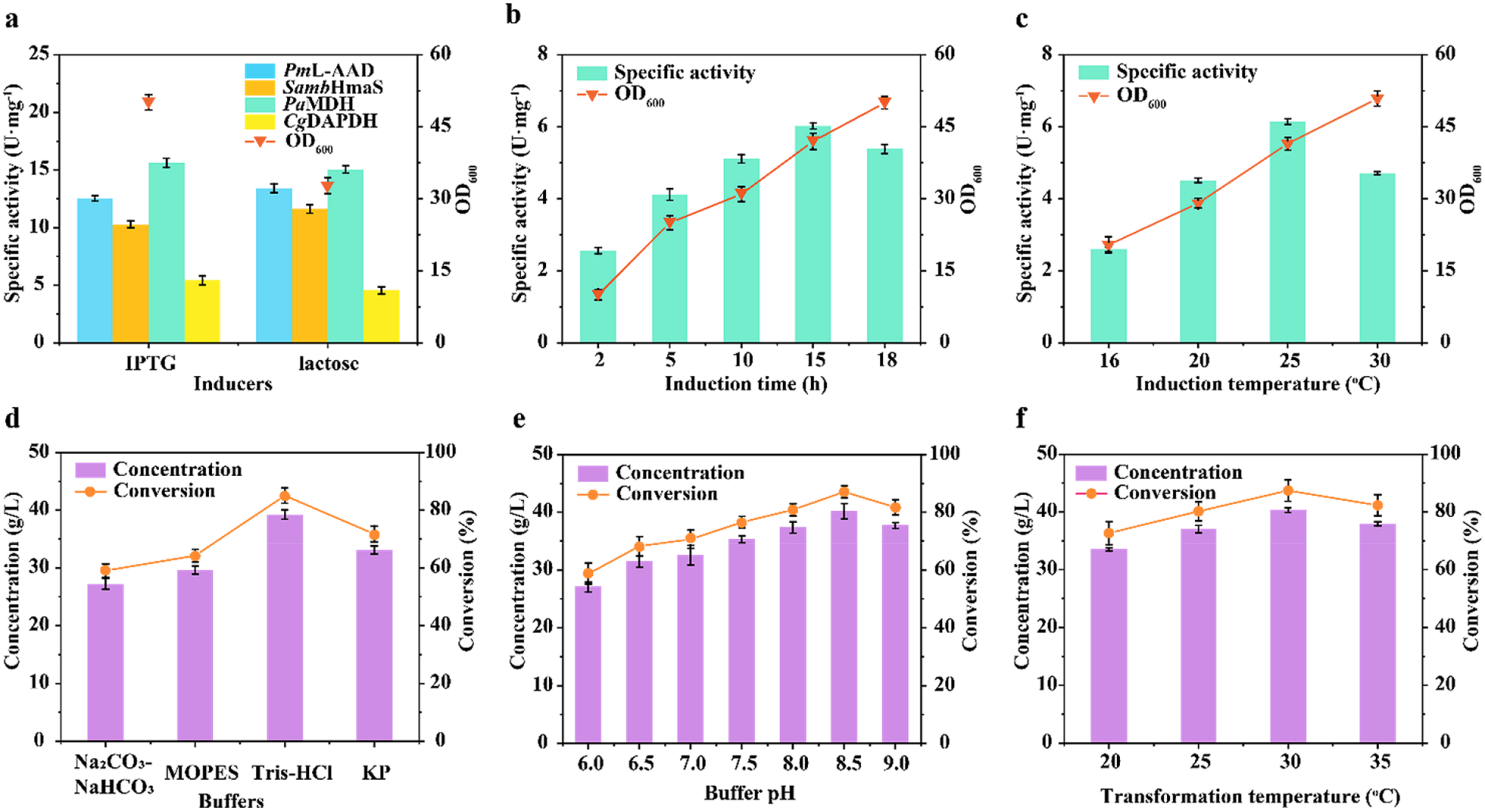
Optimizations during induction and conversion process with strain *E. coli* 07. **a** Effect of inducers (IPTG and lactose) on specific activity of the four route enzymes and cell growth of *E. coli* 07. **b** Effect of induction time on *Cg*DAPDH^BC621/D120S/W144S/I169P^ specific activity and cell growth. **c** Effect of induction temperature on *Cg*DAPDH^BC621/D120S/W144S/I169P^ specific activity and cell growth. **d** Effect of buffer type on D-HPG concentration. **e** Effect of buffer pH on D-HPG concentration. **f** Effect of transformation temperature on D-HPG concentration. Reactions were performed in triplicate with resting cells of *E. coli* 07 (20 g/L wet cells) and 50 g/L l-tyrosine in 800-mL Tris–HCl buffer (20 mM, pH 8.5, 50-g/L NH_4_Cl, 0.5-mM CoSO_4_, and 0.7-mM NADP^+^) at 500 rpm and 30 °C for 24 h. The conversion and titer were obtained after completion of the reactions and determined by HPLC analysis

The effects of inducers (IPTG and lactose) on activity and cell growth were then evaluated (Fig. 6a). Induction with IPTG (0.4-mM IPTG at 2 h) resulted in a 1.19-fold increase in activity for *Cg*DAPDH^BC621/D120S/W144S/I169P^ (5.42 ± 1.2 U·mg^−1^) and a 1.53-fold increase in cell growth (OD_600_ = 50.1 ± 1.4) relative to lactose induction; for the same IPTG concentration and induction time, the activities for *Pm*L-AAD, *Samb*HmaS, and *Pa*MDH were 12.5 ± 1.2 U mg^−1^, 10.3 ± 0.9 U·mg^−1^, and 15.6 ± 1.5 U·mg^−1^, respectively. Moreover, increase in the induction time from 2 to 15 h resulted in the highest *Cg*DAPDH^BC621/D120S/W144S/I169P^ activity (6.02 ± 0.6 U·mg^−1^), although this decreased with induction times > 15 h (Fig. 6b). Under 15-h induction by 0.4 mM IPTG, *Cg*DAPDH^BC621/D120S/W144S/I169P^ activity was further increased to 6.14 ± 0.5 U·mg^−1^ as the temperature increased from 16 °C to 25 °C, with increased cell growth also observed under these conditions [OD_600_ = 20.4 ± 0.1 (16 °C) vs. 41.5 ± 1.2 (25 °C)] (Fig. 6c). These results identified the optimal conditions as 15-h induction by IPTG at 25 °C after culturing at 37 °C for 2 h.

The effects of buffer type, pH, and temperature on the D-HPG titer at the 3-L scale were then investigated. As shown in Fig. 6d, a higher D-HPG titer was detected (39.23 ± 1.2 g/L) in Tris–HCl buffer compared with that obtained using the other three buffers tested. In Tris–HCl buffer, the D-HPG titer was further increased to a peak of 40.17 ± 0.9 g/L within a pH range of 6.0–8.5 (Fig. 6e). Assessment of the transformation temperature (range: 25–35 °C) revealed a high D-HPG titer (40.31 ± 1.1 g/L) and conversion (87.38 ± 0.6%) at 30 °C (Fig. 6f).

Under the optimal induction and transformation conditions [0.7-mM NADP^+^, 0.5-mM CoSO_4_, 20-mM Tris–HCl buffer (pH 8.5) and 30 °C], 42.69-g/L D-HPG was obtained in 3-L fermentation using 20 g/L (wet cell) *E. coli* 07 from 50-g/L l-tyrosine in 20 h with 92.5% conversion and > 99% *ee*. Finally, 32.98-g D-HPG was isolated with a 71.5% yield by preparation thin liquid chromatography, extraction, and crystallization. The identity of D-HPG was confirmed by NMR, HPLC, and HRMS analysis (Additional file 1: Fig. S11 and Fig. S12).

## Discussion

Numerous methods have been designed to efficiently produce D-HPG; these mainly include chemical synthesis and enzymatic conversion. Chemical synthesis methods include chiral separation, esterification-coupled hydrolysis, induced crystallization, and asymmetric transformation (Yu et al. 2009; Zhang et al. 2010). Although these methods are efficient for D-HPG production, their disadvantages include the need for high-cost precursors, multi-step separation and purification processes, and the production of toxic intermediates (Van et al. 2007; Zhang et al. 2015). For enzymatic conversion, a recent study used DL-HPH as a substrate in a two-step enzymatic process catalyzed by Hase and Case, resulting in 29.10-g/L D-HPG produced from 30-g/L DL-HPH in 12 h (Hu and Lin 2015); however, DL-HPH is usually obtained by the condensation of urea, phenol, and glyoxylic acid, which requires harsh conditions, thereby increasing the cost of D-HPG production (Bellini et al. 2019). Therefore, the development of a lower-cost and environmentally friendly method for efficient production of D-HPG remains a necessity. In this study, a four-enzyme cascade pathway was designed for the production of D-HPG from l-tyrosine, a low-cost and widely available amino acid. This pathway has three advantages: (1) use of a low-cost substrate and reagents (l-tyrosine and NH_4_Cl, respectively) to produce D-HPG; (2) simple procedures using a single *E. coli* strain (strain 07) for one-pot l-tyrosine conversion to D-HPG and no additional separation/purification processes; and (3) an environmentally friendly process with no generation of toxic intermediates (those generated in situ can be directly consumed in the reaction sequence).

High levels of the intermediate HPGA were generated during conversion (4.26 ± 0.04 mM) due to the lower activity of *Cg*DAPDH^BC621^, which caused an imbalance in enzyme activity in the cascade. Therefore, a mechanism-guided “conformation rotation” strategy was applied to shorten the d_(C6HDAP−C4NNADP)_ in variant *Cg*DAPDH^BC621/D120S/W144S/I169P^, resulting in 37-fold and 119-fold increases in the specific activity and *k*_cat_/*K*_m_ value relative to those of *Cg*DAPDH^BC621^, respectively. To improve the catalytic properties of DAPDH, previous studies employed several protein engineering strategies, including random mutagenesis and rational design (Akita et al. 2018; Cheng et al. 2018; Zhang et al. 2018). Random mutagenesis can optimize enzyme efficiency without the need for detailed knowledge of the protein structure (Cho et al. 2019). *Cg*DAPDH^BC621^ was originally obtained after screening ~ 100,000 variants, which exhibited a 975-fold increase in specific activity toward d-2-aminooctanoate (Vedha et al. 2006). However, random mutation might not cover all sequences and requires enormous screening effort (Chen et al. 2019). In contrast, rational design is based on analysis of structure–function relationships or catalytic mechanisms, thereby greatly reducing screening efforts (Kan et al. 2016). Recently, this approach was used to identify a double-mutant variant of *Symbiobacterium thermophilum* (*St*DAPDH^W121L/H227I^) via structural alignment, resulting in a 34.45-fold increase in activity toward 2-oxo-4-phenylbutyric acid relative to that of wild-type *St*DAPDH (Cheng et al. 2018). However, existing rational-design strategies mainly focus on the DAPDH active site, with few strategies addressing the asymmetric amination mechanism of DAPDH. Our “conformation rotation” strategy had three main characteristics: (1) the rate-limiting step was defined based on the reaction mechanism (the hydride-transfer distance [d_(C6HDAP−C4NNADP)_]) (Fig. 4a) and was appropriately modified to promote efficient asymmetric amination of HPGA; (2) specific hotspots were defined as bulky residues proximal to the D-HPG substrate and were used to perform rational engineering of beneficial variants; and (3) a few variants were created, which was more efficient than random mutagenesis and enabled rapid identification of optimal variants.

By introducing *Cg*DAPDH^BC621/D120S/W144S/I169P^ into strain *E. coli* 07 and then optimizing the induction and transformation conditions, 42.69 g/L of D-HPG was obtained with 92.5% conversion, 71.5% isolated yield, and > 99% *ee* during one-pot transformation. Compared with the highest D-HPG titer reported to date (29.10 g/L) (Table 4), the use of *E. coli* 07 for the enzymatic production of D-HPG can increase the titer by 49.7%. These findings demonstrate the efficacy of the developed cascade pathway for improving the D-HPG titer, and represents a potentially attractive strategy for the industrial production of D-HPG.

**Table 4:** Comparison of D-HPG production via enzymatic process

Strains	Culture condition	D-HPG titer (g/L)	Conversion (%)	Isolated yield (%)	*ee*	References
*Pseudomonas putida*	Bioconversion^a^	5.06	60.5	n/a	n/a	(Nandanwar et al. 2013)
*Bacillus subtilis*	Chemoenzymatic Synthesis	14.32	95	80	n/a	(Li et al. 2019)
*Ralstonia pickettii*	Bioconversion^a^	25.07	94	n/a	n/a	(Yu et al. 2009)
*Escherichia coli*	Bioconversion^a^	23.40	100	n/a	n/a	(Liu et al. 2019)
*Escherichia coli*	Bioconversion^a^	29.10	97.0	88	n/a	(Hu and Lin 2015)
*Escherichia coli*	Bioconversion^b^	42.69	92.5	71.5	> 99	This study

n/a: not available

^a^ Dual-enzyme cascade employing Hase and Case

^b^ Four-enzyme cascade in this study

## Conclusions

To develop an efficient method for D-HPG production, a four-enzyme cascade pathway using l-tyrosine as a substrate was designed and the pathway was reconstructed in vivo. The efficiency of the pathway was further increased by improving the catalytic activity of *Cg*DAPDH, the rate-limiting step, toward the HPGA intermediate using a mechanism-guided “conformation rotation” strategy. Introduction of the best engineered variant (CgDAPDH^BC621/D120S/W144S/I169P^) into *E. coli* 07 allowed one-pot conversion of l-tyrosine to obtain 42.69-g/L D-HPG, 92.5% conversion, 71.5% isolated yield, and > 99% *ee* during 3-L fermentation. These results describe a potential enzymatic process that allows for the industrial-scale production of D-HPG from cheap amino acids.

### Supplementary Information

**Additional file 1: Table S1.** Primers used for variants construction. **Table S2.** L-AAD specific enzyme activities from different organisms. **Table S3.** HmaS specific enzyme activities from different organisms. **Table S4.** MDH specific enzyme activities from different organisms. **Table S5.** DAPDH specific enzyme activities from different organisms. **Table S6.** Screening of the NNK-based site-saturation mutagenesis depending on the formazan-based high-throughput methods. **Table S7.** Genetic information used for pathway construction. **Table S8.** PCR amplification system. **Table S9.** Primers used for genetic construction. **Fig. S1.** Verify 3D of *Cg*DAPDH^BC621^ structure. **Fig. S2.** Ramachandran plot of *Cg*DAPDH^BC621^ structure. **Fig. S3.** The homology model of *Cg*DAPDH^BC621^ optimized by dynamic simulation. **Fig. S4.** NMR spectra of D-HPG. **Fig. S5.** SDS-PAGE analysis of engineered *E. coli* 02-06 expressing *PaMDH* and *Cg*DAPDH^BC621^. **Fig. S6.** Improvement of the specific activity of *Cg*DAPDH^BC621^ toward HPGA by NNK-based site-saturation mutagenesis. **Fig. S7.** Improvement of the specific activity of *Cg*DAPDH^BC621^ toward HPGA by recombinant mutagenesis. **Fig. S8.** Improvement of the specific activity of *Cg*DAPDH^BC621^ toward HPGA by iterative saturation mutagenesis. **Fig. S9.** The hydrogen bond distances between D-HPG and the residues in binding cavity. **Fig. S10.** Effect of the best variant *Cg*DAPDH^BC621/D120S/W144S/I169P^ on D-HPG production. **Fig. S11.** Chiral HPLC chromatograms of HPG. **Fig. S12.** Identity of isolated product.

## Data Availability

All data generated or analyzed during this study are included in this article.

## Abbreviations

D-HPG: d-*p*-Hydroxyphenylglycine
HPGA: 4-Hydroxyphenylglyoxylic acid
DL-HPG: dl-*p*-hydroxyphenylglycine
DL-HPH: dl-hydroxyphenylhydantoin
Hase: d-hydantoinase
Case: *N*-Carbamoyl-d-amino-acid hydrolase
L-HPG: l-*p*-hydroxyphenylglycine
D-Phg: d-phenylglycine
L-Phg: l-phenylglycine
Hmo: 4-Hydroxymandelate oxidase
HpgT: (*S*)-3, 5-Dihydroxyphenylglycine transaminase
HpgAT: D-4-Hydroxyphenylglycine transaminase
PMS: Phenazine methosulfate
NBT: Nitro blue tetrazolium
DAPDH: *meso*-Diaminopimelate dehydrogenase
HPP: 4-Hydroxyphenylpyruvate
L-AAD: l-amino acid deaminase
(*S*)-HMA: (*S*)-4-Hydroxymandelate
HmaS: 4-Hydroxymandelate synthase
MDH: (*S*)-Mandelate dehydrogenase
DCW: Dry cell weight
