# Supplementary material for: A multi-enzyme cascade for efficient production of d-p-hydroxyphenylglycine from l-tyrosine
Source: Bioresour Bioprocess. 2021 May 21;8(1):41. doi: 10.1186/s40643-021-00394-2 (PMC10991500; doi:10.1186/s40643-021-00394-2)
Supplement: Supplementary file 1 — Additional file 1: Table S1. Primers used for variants construction. Table S2. L-AAD specific enzyme activities from different organisms. Table S3. HmaS specific enzyme activities from different organisms. Table S4. MDH specific enzyme activities from different organisms. Table S5. DAPDH specific enzyme activities from different organisms. Table S6. Screening of the NNK-based site-saturation mutagenesis depending on the formazan-based high-throughput methods. Table S7. Genetic information used for pathway construction. Table S8. PCR amplification system. Table S9. Primers used for genetic construction. Fig. S1. Verify 3D of CgDAPDHBC621 structure. Fig. S2. Ramachandran plot of CgDAPDHBC621 structure. Fig. S3. The homology model of CgDAPDHBC621 optimized by dynamic simulation. Fig. S4. NMR spectra of D-HPG. Fig. S5. SDS-PAGE analysis of engineered E. coli 02-06 expressing PaMDH and CgDAPDHBC621. Fig. S6. Improvement of the specific activity of CgDAPDHBC621 toward HPGA by NNK-based site-saturation mutagenesis. Fig. S7. Improvement of the specific activity of CgDAPDHBC621 toward HPGA by recombinant mutagenesis. Fig. S8. Improvement of the specific activity of CgDAPDHBC621 toward HPGA by iterative saturation mutagenesis. Fig. S9. The hydrogen bond distances between D-HPG and the residues in binding cavity. Fig. S10. Effect of the best variant CgDAPDHBC621/D120S/W144S/I169P on D-HPG production. Fig. S11. Chiral HPLC chromatograms of HPG. Fig. S12. Identity of isolated product. [file 40643_2021_394_MOESM1_ESM.docx]

**A multi-enzyme cascade for efficient production of D-*p*-hydroxyphenylglycine from L-tyrosine**

Xu Tan^1, 2^, Sheng Zhang^3^, Wei Song^1, 2^, Jia Liu^2^, Cong Gao^2^, Xiulai Chen^2^, Liming Liu^2^, Jing Wu^1^*

^1^ School of Pharmaceutical Science, Jiangnan University, Wuxi 214122, China

^2^ State Key Laboratory of Food Science and Technology, Jiangnan University, Wuxi 214122, China

^3^ Zhejiang Tianrui Chemical Co., Ltd, Quzhou 324400, China

*** Corresponding author.**

School of Pharmaceutical Science, Jiangnan University, 1800 Lihu Road, Wuxi 214122, China.

Fax/Tel.: +86-510-85915657

E-mail: wujing@jiangnan.edu.cn

This doc file includes:

Supplementary Table 1 to Table 9

Supplementary Figure 1 to Figure 12

Supplementary materials and methods

# Supplementary Tables

**Table S1.** Primers used for variants construction

| Primer | Sequence (5’-3’) |
| --- | --- |
| W119-S | TGTCTCCACCGGCNNKGATCCAGGAATGT |
| W119-A | ACATTCCTGGATCMNNGCCGGTGGAGACA |
| D120-S | CTCCACCGGCTGGNNKCCAGGAATGTTCT |
| D120-A | AGAACATTCCTGGMNNCCAGCCGGTGGAG |
| W144-S | GCAGCACACCTTCNNKGGCCCAGGTTTGT |
| W144-A | ACAAACCTGGGCCMNNGAAGGTGTGCTGC |
| L150-S | CCCAGGTTTGTCANNKGGCCACTCCGGCG |
| L150-A | CGCCGGAGTGGCCMNNTGACAAACCTGGG |
| G151-S | CAGGTTTGTCACTGNNKCACTCCGGCGCT |
| G151-A | AGCGCCGGAGTGMNNCAGTGACAAACCTG |
| H152-S | TTTGTCACTGGGCNNKTCCGGCGCTTTGC |
| H152-A | GCAAAGCGCCGGAMNNGCCCAGTGACAAA |
| I169-S | GGCCGTCCAGTACNNKCTCCCATCCGAAG |
| I169-A | CTTCGGATGGGAGMNNGTACTGGACGGCC |
| Y223-S | CTACTTCGTTGGCNNKGAAGTCGAAGTGA |
| Y223-A | TCACTTCGACTTCMNNGCCAACGAAGTAG |
| N270-S | CAAGCTGGACCGANNKCCAGATTTCACCG |
| N270-A | CGGTGAAATCTGGMNNTCGGTCCAGCTTG |
| D120S-S | CTCCACCGGCTGGAGCCCAGGAATGTTCT |
| D120S-A | AGAACATTCCTGGGCTCCAGCCGGTGGAG |
| Y223C-S | CTACTTCGTTGGCTGTGAAGTCGAAGTGA |
| Y223C-A | TCACTTCGACTTCACAGCCAACGAAGTAG |

**Table S2.** L-AAD specific enzyme activities from different organisms

| Enzymes | Organisms | Specific activity^a^  (U·mg^-1^·protein) |
| --- | --- | --- |
| *Pv*L-AAD | *Proteus vulgaris* | 7.39±0.19 |
| *Pm*L-AAD | *Proteus mirabilis* | 8.95±0.38 |

*Note.* L-AAD: L-amino acid deaminase.

^a^ The specific activity was determined with 10 μM purified L-AAD and 10 mM L-tyrosine in 1 ml Tris-HCl buffer (50 mM, pH 8.0) at 30 °C for 15 min.

**Table S3.** HmaS specific enzyme activities from different organisms

| Enzymes | Organisms | Specific activity^a^  (U·mg^-1^·protein) |
| --- | --- | --- |
| *Ao*HmaS | *Amycolatopsis orientalis* | 6.09±0.75 |
| *Sco*HmaS | *Streptomyces coelicolor* | 3.18±0.97 |
| *Samb*HmaS | *Streptomyces ambofaciens* | 7.25±0.98 |
| *Mau*HmaS | *Micromonospora aurantiaca* | 0.68±0.08 |
| *Sro*HmaS | *Streptosporangium roseum* | n/a |

*Note.* HmaS: 4-hydroxymandelate synthase; n/a, not available.

^a^ The specific activity was determined with 10 μM purified HmaS, 10 mM HPP, and 0.5 mM CoSO_4_ in 1 ml Tris-HCl buffer (50 mM, pH 8.0) at 30 °C for 15 min.

**Table S4.** MDH specific enzyme activities from different organisms

| Enzymes | Organisms | Specific activity^a^  (U·mg^-1^·protein) |
| --- | --- | --- |
| *Hel*MDH | *Halomonas elongata* | 0.24±0.07 |
| *Bgl*MDH | *Burkholderia gladioli* | 2.35±0.21 |
| *Cs*MDH | *Chromohalbacter salexigens* | 5.33±0.87 |
| *Ppu*MDH | *Pseudomonas putida* | 5.69±0.34 |
| *Sp*MDH | *Sphingobium* sp*.* | 4.37±0.61 |
| *Cb*MDH | *Cupriavidus basilensis* | 0.04±0.02 |
| *Pa*MDH | *Pseudomonas aeruginosa* | 9.26±0.37 |

*Note.* MDH: (*S*)-mandelate dehydrogenase.

^a^ The specific activity was determined with 10 μM purified MDH 10 mM (*S*)-HMA, and 0.5 mM NADP^+^ in 1 ml Tris-HCl buffer (50 mM, pH 8.0) at 30 °C for 15 min.

**Table S5.** DAPDH specific enzyme activities from different organisms

| Enzymes | Organisms | Specific activity^a^  (U·mg^-1^·protein) |
| --- | --- | --- |
| *St*DAPDH | *Symbiobacterium thermophilum* | 0.09±0.40 |
| *Cg*DAPDH | *Corynebacterium glutamicum* | n/a |
| *Cg*DAPDH^BC621^ | *Corynebacterium glutamicum* | 0.14±1.60 |
| *Bf*DAPDH | *Bacteroides fragilis* | n/a |
| *Pv*DAPDH | *Proteus vulgaris* | n/a |
| *Pm*DAPDH | *Proteus mirabilis* | n/a |

*Note.* DAPDH: *meso*-diaminopimelate dehydrogenase; n/a, not available.

^a^ The specific activity was determined with 10 μM purified DAPDH, 10 mM HPGA, 0.5 mM NADPH and 20 mM NH_4_Cl in 1 ml Tris-HCl buffer (50 mM, pH 8.0) at 30 °C for 15 min.

**Table S6.** Screening of the NNK-based site-saturation mutagenesis depending on the formazan-based high-throughput methods

| Mutants | The lowest ratio of absorbance value | Variant |
| --- | --- | --- |
| *Cg*DAPDH^BC621^ | 1 | / |
| *Cg*DAPDH^BC621/W119^ | 1.19 | / |
| *Cg*DAPDH^BC621/D120S^ | 0.70 | D120S |
| *Cg*DAPDH^BC621/W144^ | 0.83 | / |
| *Cg*DAPDH^BC621/L150^ | 1.06 | / |
| *Cg*DAPDH^BC621/G151^ | 0.94 | / |
| *Cg*DAPDH^BC621/H152^ | 1.03 | / |
| *Cg*DAPDH^BC621/I169P^ | 0.62 | I169P |
| *Cg*DAPDH^BC621/I169Y^ | 0.62 | I169Y |
| *Cg*DAPDH^BC621/N270^ | 0.95 | / |
| *Cg*DAPDH^BC621/Y223C^ | 0.77 | Y223C |

Ratio of absorbance value =absorbance value of mutants/absorbance value of *Cg*DAPDH^BC621^

**Table S7.** Genetic information used for pathway construction

| Enzyme | GenBank | Organism | Culture collection center |  |
| --- | --- | --- | --- | --- |
| *Pv*L-AAD | MK258171 | *Proteus vulgaris* | GenScript |  |
| *Pm*L-AAD | U35383 | *Proteus mirabilis* | GenScript |  |
| *Mau*HmaS | ADL47009 | *Micromonospora aurantiaca* | CGMCC 4.2103 |  |
| *Ao*HmaS | ANN17105 | *Amycolatopsis orientalis* | CGMCC 4.6521 |  |
| *Sco*HmaS | 1098663 | *Streptomyces coelicolor* | CICC 11017 |  |
| *Sro*HmaS | ACZ89671 | *Streptosporangium roseum* | CGMCC 4.2071 |  |
| *Samb*HmaS | AKZ58697 | *Streptomyces ambofaciens* | CGMCC 4.1528 |  |
| *Hel*MDH | CBV41027 | *Halomonas elongata* | CGMCC 1.6329 |  |
| *Bgl*MDH | AJW98399 | *Burkholderia gladioli* | ATCC 10248 |  |
| *Cs*MDH | ABE58432 | *Chromohaloacter salexigens* | ATCC BAA-138 |  |
| *Ppu*MDH | BAN56662 | *Pseudomonas putida* | CICC 10298 |  |
| *Sp*MDH | BBD01499 | *Sphingobium* sp. | CICC 10686 |  |
| *Cb*MDH | CP010537 | *Cupriavidus basilensis* | CICC 24102 |  |
| *Pa*MDH | AGM49308 | *Pseudomonas aeruginosa* | CICC 20236 |  |
| *St*DAPDH | BAD40410 | *Symbiobacterium thermophilum* | GenScript |  |
| *Cg*DAPDH | CAF21279 | *Corynebacterium glutamicum* | CICC 10226 |  |
| *Bf*DAPDH | AKA53147 | *Bacteroides fragilis* | CICC 24309 |  |
| *Pv*DAPDH | ATM98278 | *Proteus vulgaris* | CICC 21599 |  |
| *Pm*DAPDH | CAR40477 | *Proteus mirabilis* | CGMCC 1.1807 |  |

**Table S8.** PCR amplification system

| Component | Volume (μL) |
| --- | --- |
| 5×PS Buffer | 20 |
| dNTPs | 8 |
| Primer 1 | 0.5 |
| Primer 2 | 0.5 |
| PrimeSTAR®HS | 1 |
| Template | 2 |
| ddH_2_O | 68 |
| Total | 100 |

**Table S9.** Primers used for genetic construction

| Primer | Sequence (5’-3’) |
| --- | --- |
| *Mau*HmaS-S | GTGGTGGTGGTGGTGCTCGAGTCATGCCCCAACCCCTCC |
| *Mau*HmaS-A | ATGGGTCGCGGATCCGAATTCATGACCATCAACGGGATAGATCA |
| *Ao*HmaS-S | GTGGTGGTGGTGGTGCTCGAGTCATCGCCGAGCGGCGCC |
| *Ao*HmaS-A | AGCAAATGGGTCGCGGATCCATGCAGAATTTCGAGATCGACTAC |
| *Sco*HmaS-S | GTGGTGGTGGTGGTGCTCGAGTCATCGGCCGGCCACTTC |
| *Sco*HmaS-A | CAGCAAATGGGTCGCGGATCCGTGCTCCCTCCTTTCCCCT |
| *Sro*HmaS-S | GTGGTGGTGGTGGTGCTCGAGTCAGGCGGCGGCCTCTTC |
| *Sro*HmaS-A | ATGGGTCGCGGATCCGAATTCATGGCCTCGTCAGGAACGC |
| *Samb*HmaS-S | GTGGTGGTGGTGGTGCTCGAGTCACCTGGACCAGCCGTTC |
| *Samb*HmaS-A | ATGGGTCGCGGATCCGAATTCATGACCCATGCCCCGAAC |
| *Hel*MDH-S | GTGGTGGTGGTGGTGCTCGAGTCAACGGCCGCGGTGTCG |
| *Hel*MDH-A | ATGGGTCGCGGATCCGAATTCGTGAAACGCCGTCCCTATGC |
| *Bgl*MDH-S | GTGGTGGTGGTGGTGCTCGAGTCACCAGCCTTCGCCGGG |
| *Bgl*MDH-A | ATGGGTCGCGGATCCGAATTCATGCCGCCACCGATCAAC |
| *Cs*MDH-S | GTGGTGGTGGTGCTCGAGCTAGGCGGGGCTAACCG |
| *Cs*MDH-A | TGGGTCGCGGATCCGAATTCATGAAAAGAAGCATGCCGC |
| *Ppu*MDH-S | TGGTGGTGGTGGTGGTGCTCGAGTCATGCGTGTGTTCCTTTACC |
| *Ppu*MDH-A | TGGGTCGCGGATCCGAATTCATGAGCCAGAATCTCTTTAACGTT |
| *Sp*MDH-S | GGTGGTGGTGCTCGAGTCAGATGGGGGCTGCCGGCATGA |
| *Sp*MDH-A | TGGGTCGCGGATCCGAATTCGTGGCTGCCAGCTGCGACG |
| *Cb*MDH-S | GGTGGTGGTGGTGCTCGAGTCATAGCGAATTCCGGATG |
| *Cb*MDH-A | TCGCGGATCCGAATTCATGTGGTGCAGCGCGAGCAC |
| *Pa*MDH-S | GTGGTGGTGGTGGTGCTCGAGTCATGCGTGTGTTCCTTTACCA |
| *Pa*MDH-A | TGGGTCGCGGATCCGAATTCATGAGCCAGAATCTCTTTAACGTT |
| *Cg*DAPDH-S | TAAGAAGGAGATATACCATGGGCATGACCAACATCCGCG |
| *Cg*DAPDH-A | GTGGTGGTGGTGGTGCTCGAGTTAGACGTCGCGTGCGATC |
| *Bf*DAPDH-S | TAAGAAGGAGATATACCATGGGCATGAAAAAAGTAAGAGCAGCC |
| *Bf*DAPDH-A | GTGGTGGTGGTGGTGCTCGAGTTATACCAGGTGGCCGATCCA |
| *Pv*DAPDH-S | TAAGAAGGAGATATACCATGGGCATGAATACTAAAATAAAAGTAGCCATT |
| *Pv*DAPDH-A | TTGTCGACGGAGCTCGAATTCTTAAACCAATTTGGCAATTAATGTATT |
| *Pm*DAPDH-S | TAAGAAGGAGATATACCATGGGCATGAATACTAAAATAAAAGTCGCTATT |
| *Pm*DAPDH-A | TTGTCGACGGAGCTCGAATTCTTACACCAGTTTTGCGATTAATGTAT |

# Supplementary Figures


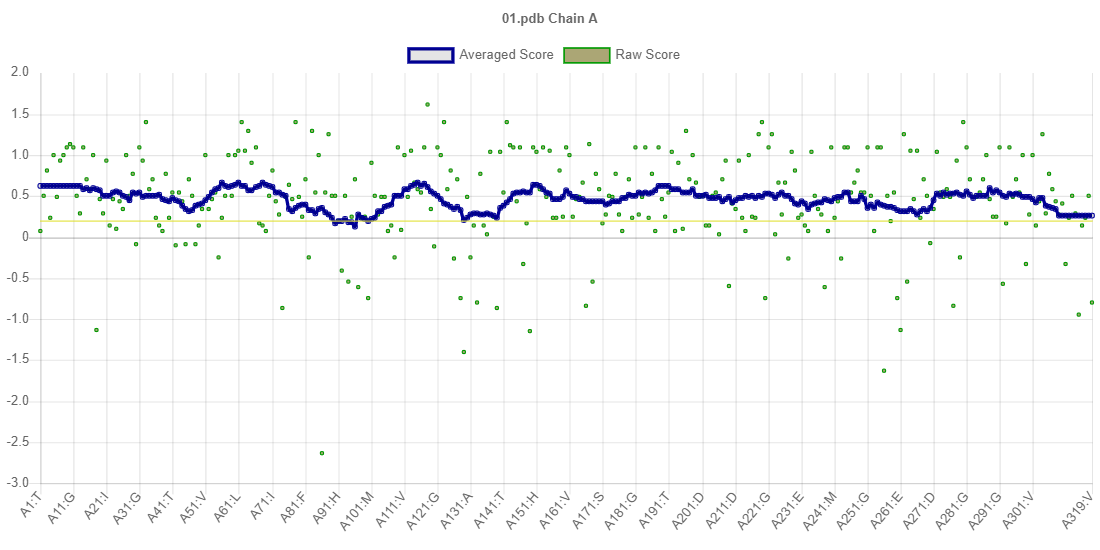


**Fig. S1.** Verify 3D of *Cg*DAPDH^BC621^ structure.


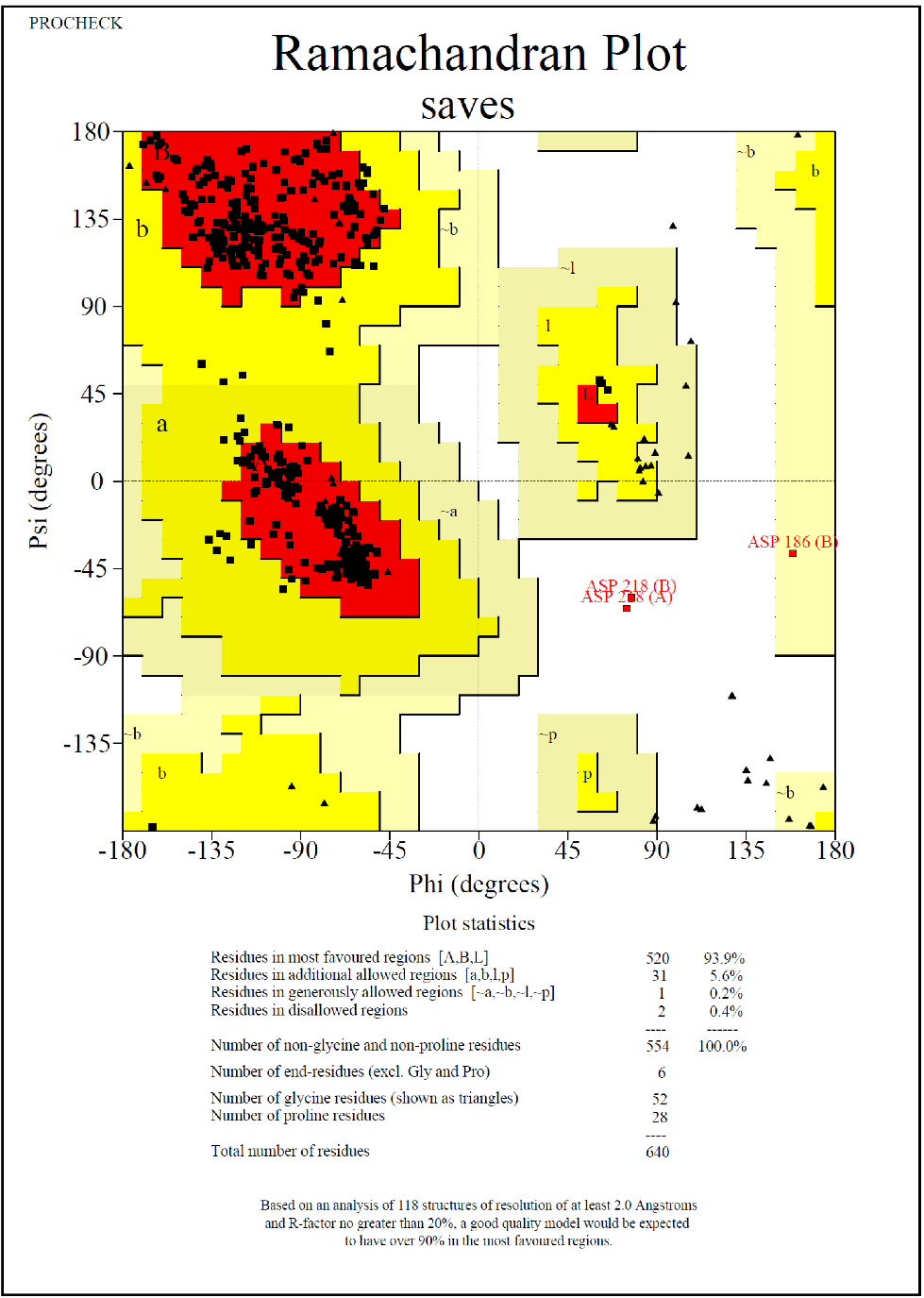


**Fig. S2.** Ramachandran plot of *Cg*DAPDH^BC621^ structure.


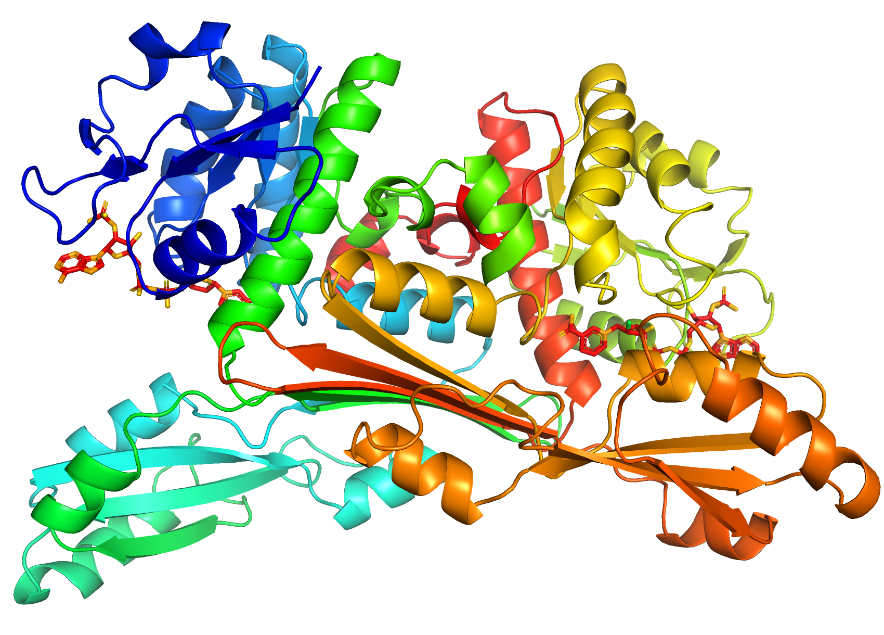


**Fig. S3.** The homology model of *Cg*DAPDH^BC621^ optimized by dynamic simulation.


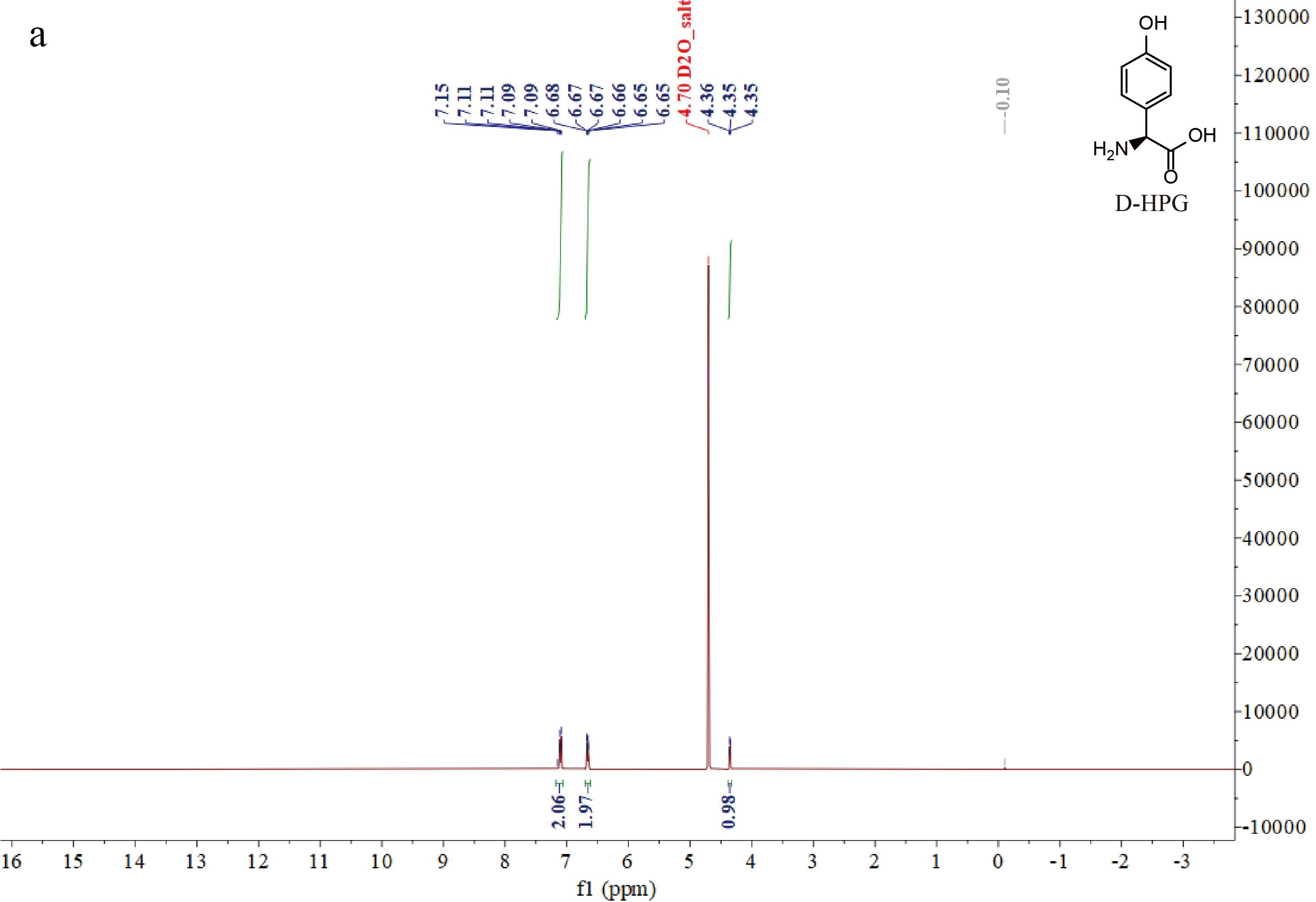

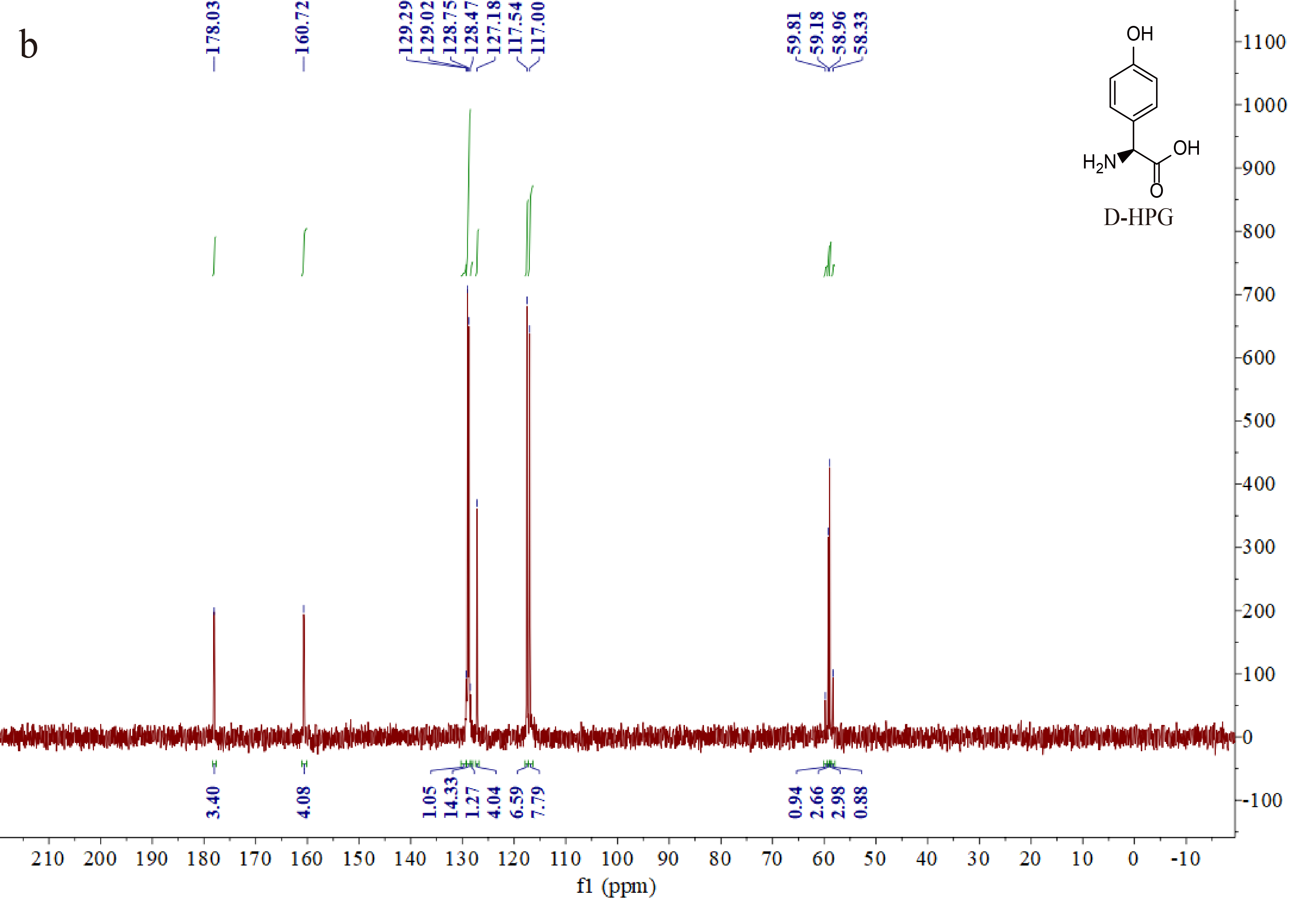


**Fig. S4.** NMR spectra of D-HPG. (a) ^1^H-NMR spectra of D-HPG. (b) ^13^C-NMR spectra of D-HPG.

**
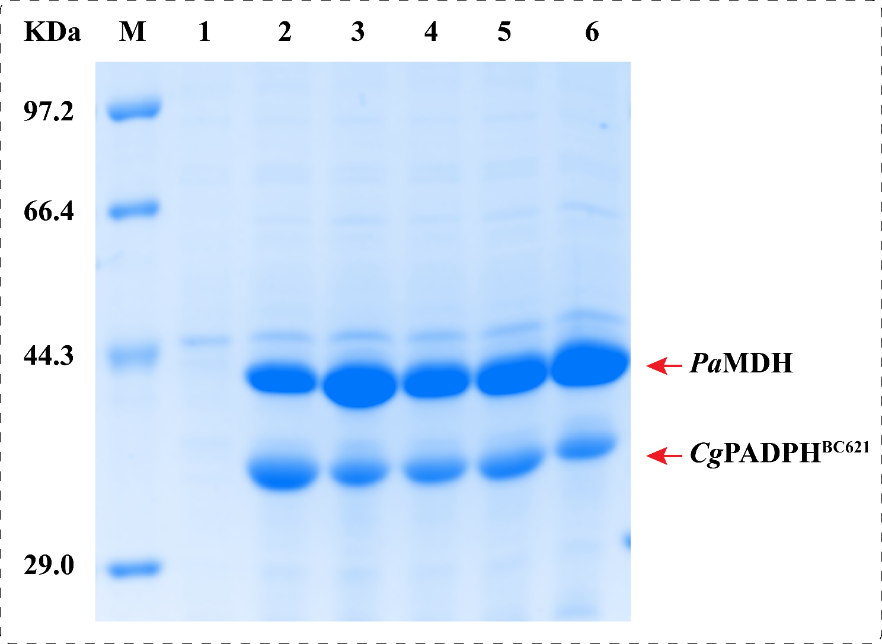
**

**Fig. S5.** SDS-PAGE analysis of engineered *E. coli* 02-06 expressing *Pa*MDH and *Cg*DAPDH^BC621^. M: Maker; Line 1: *E. coli* BL21 without overexpressing any enzymes. Line 2: *E. coli* 02; Line 3: *E. coli* 03; Line 4: *E. coli* 04; Line 5: *E. coli* 05; Line 6: *E. coli* 06.

**
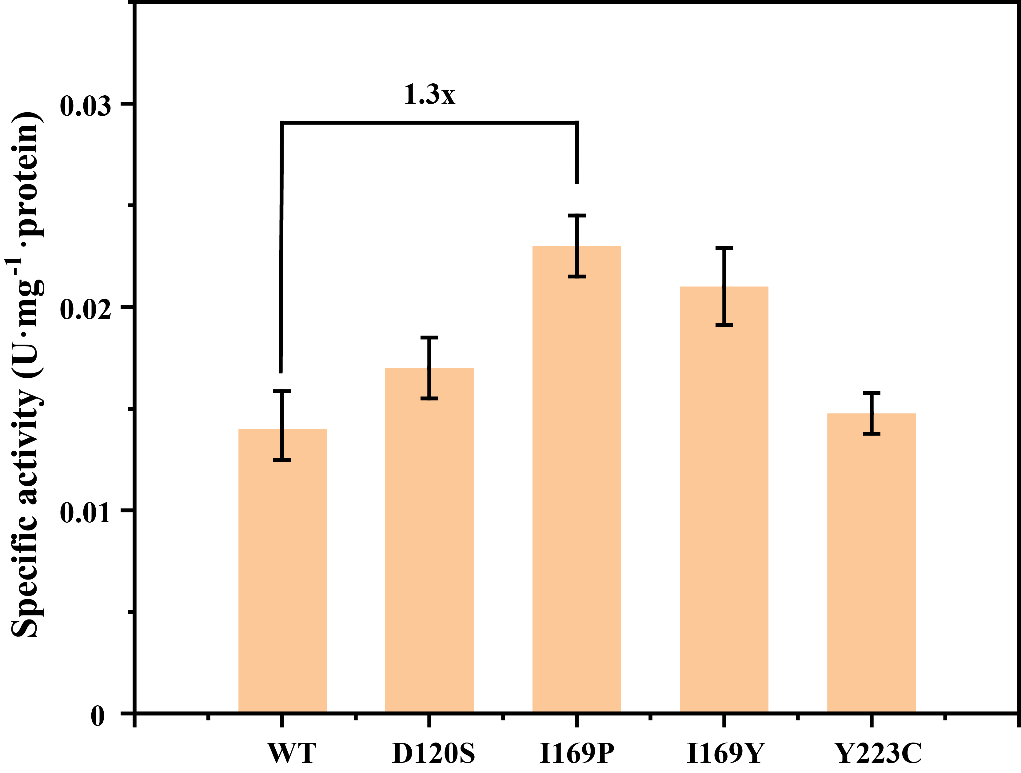
**

**Fig. S6.** Improvement of the specific activity of *Cg*DAPDH^BC621^ toward HPGA by NNK-based site-saturation mutagenesis.


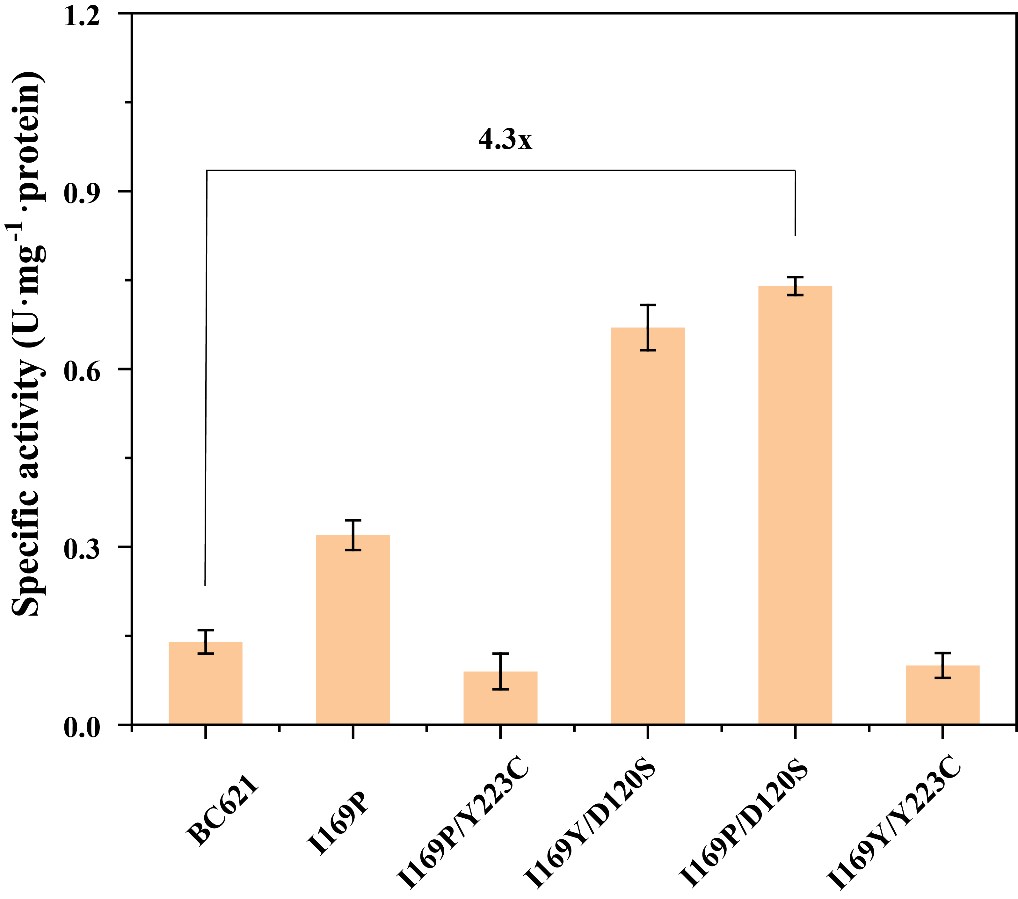


**Fig. S7.** Improvement of the specific activity of *Cg*DAPDH^BC621^ toward HPGA by recombinant mutagenesis.


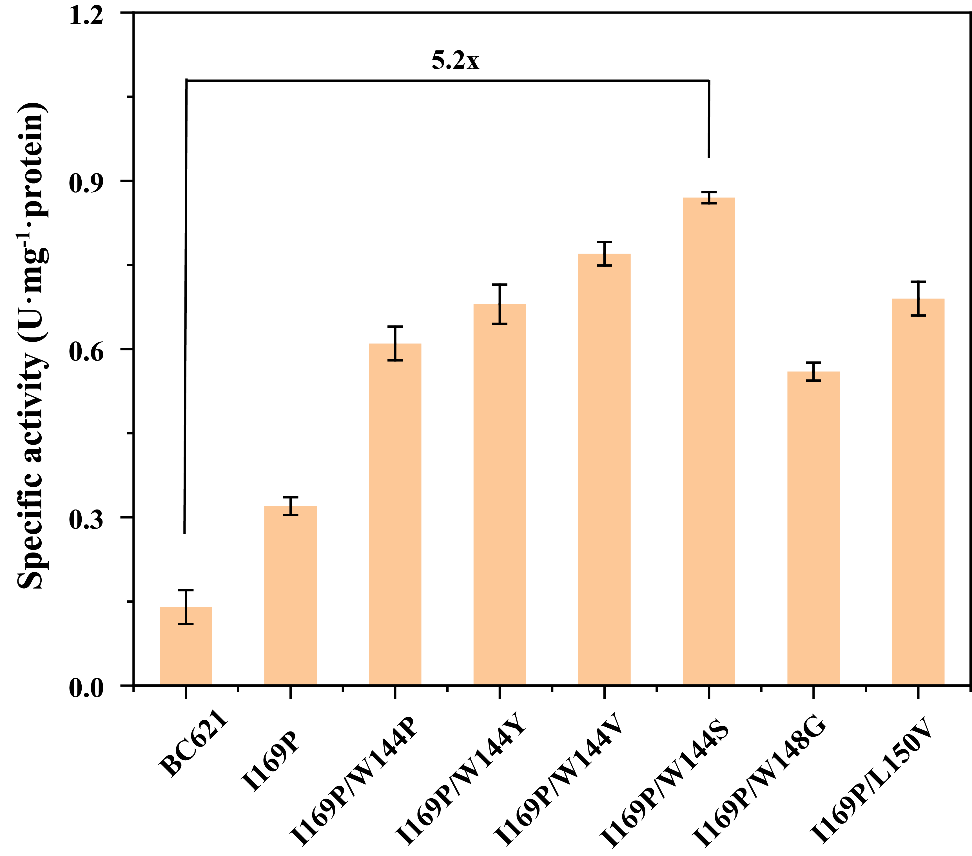


**Fig. S8.** Improvement of the specific activity of *Cg*DAPDH^BC621^ toward HPGA by iterative saturation mutagenesis.


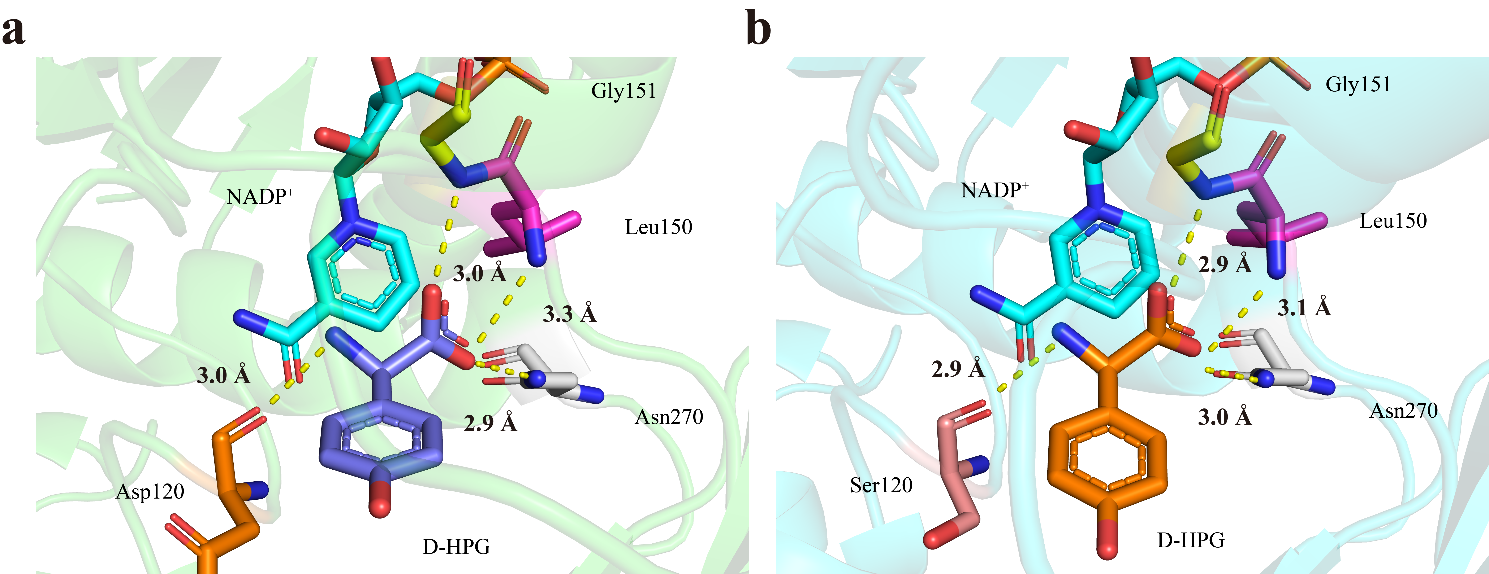


**Fig. S9.** The hydrogen bond distances between D-HPG and the residues in binding cavity. (a) The hydrogen-bond interactions between D-HPG and the residues of *Cg*DAPDH^BC621^. (b) The hydrogen-bond interactions between D-HPG and the residues of *Cg*DAPDH^BC621/D120S/W144S/I169P^. The hydrogen-bond interactions are shown in yellow dash line. D-HPG is shown in purple and orange in *Cg*DAPDH^BC621^ and *Cg*DAPDH^BC621/D120S/W144S/I169P^, respectively; NADP^+^ cofactor is shown in cyan, residue D120 and S120 are shown in orange and lightpink, respectively, L150 is shown in magenta, G151 is shown in yellow, and N270 is shown in white.


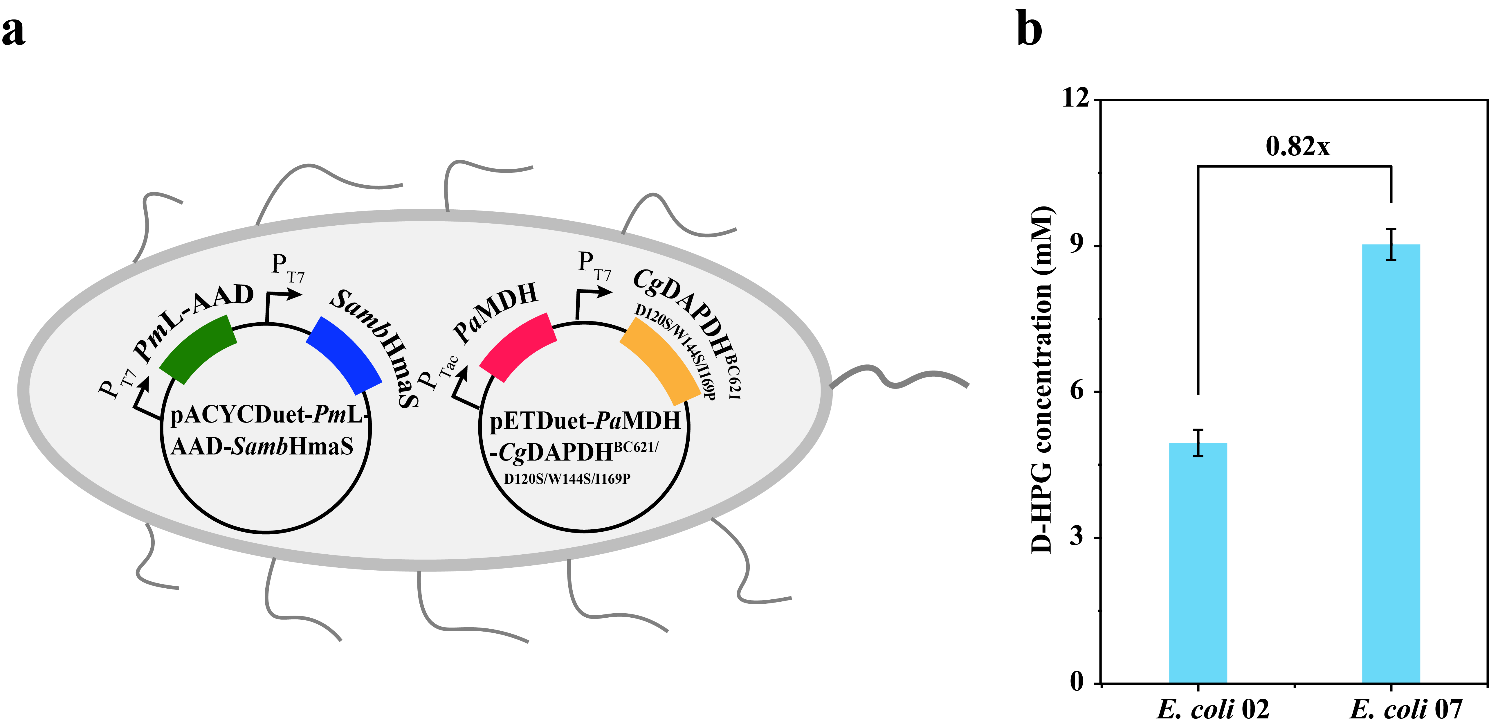


**Fig. S10.** Effect of the best variant *Cg*DAPDH^BC621/D120S/W144S/I169P^ on D-HPG production. (a) Strain *E. coli* 07 containing double plasmids to express *Pm*L-AAD, *Samb*HmaS, *Pa*MDH, and *Cg*DAPDH^BC621/D120S/W144S/I169P^. (b) Improvement of D-HPG titer with *E. coli* 07 as biocatalyst.


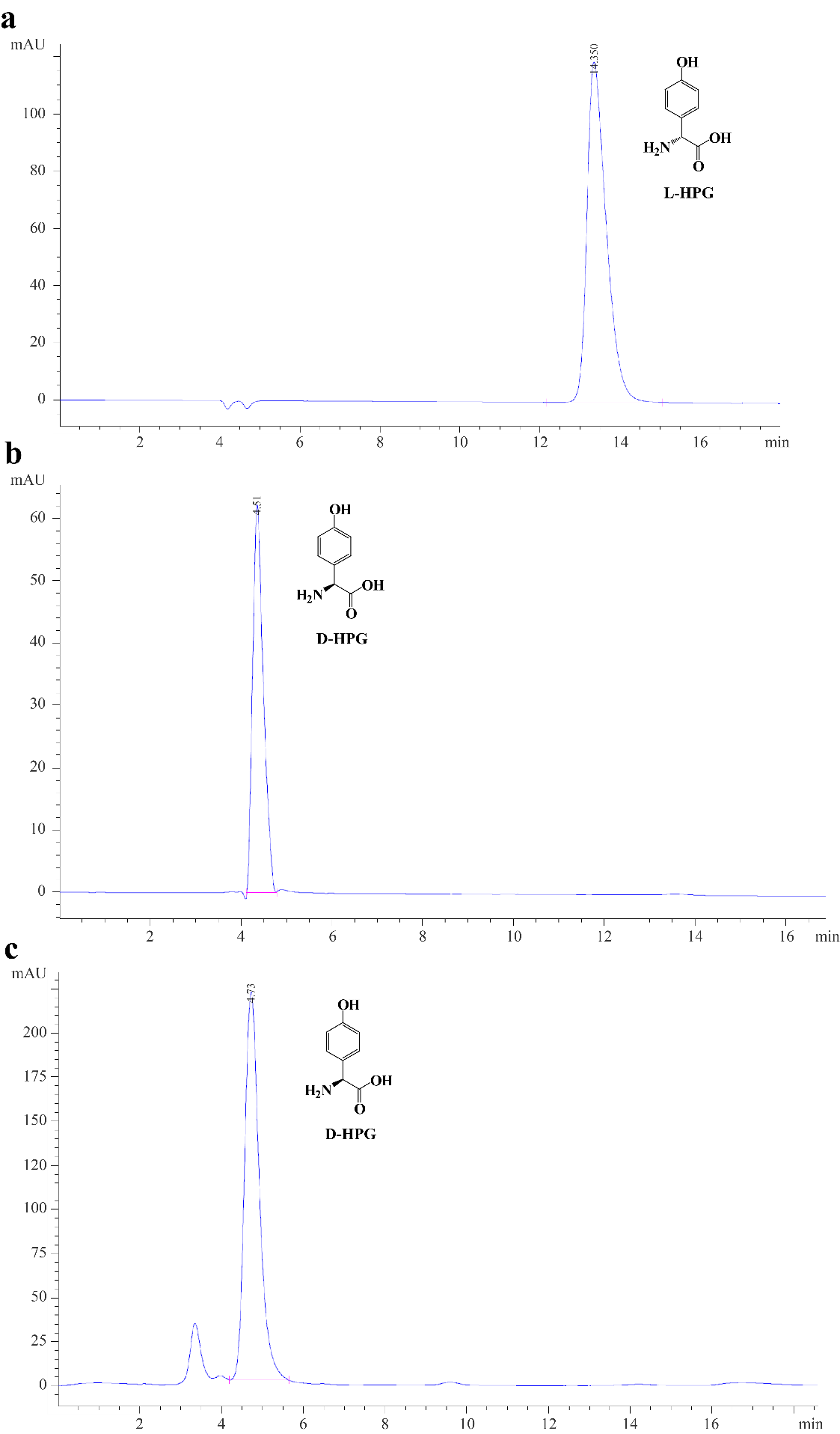


**Fig. S11.** Chiral HPLC chromatograms of HPG. (a) L-HPG standard. (b) D-HPG standard. (c) Sample from biotransformation of L-tyrosine to D-HPG.


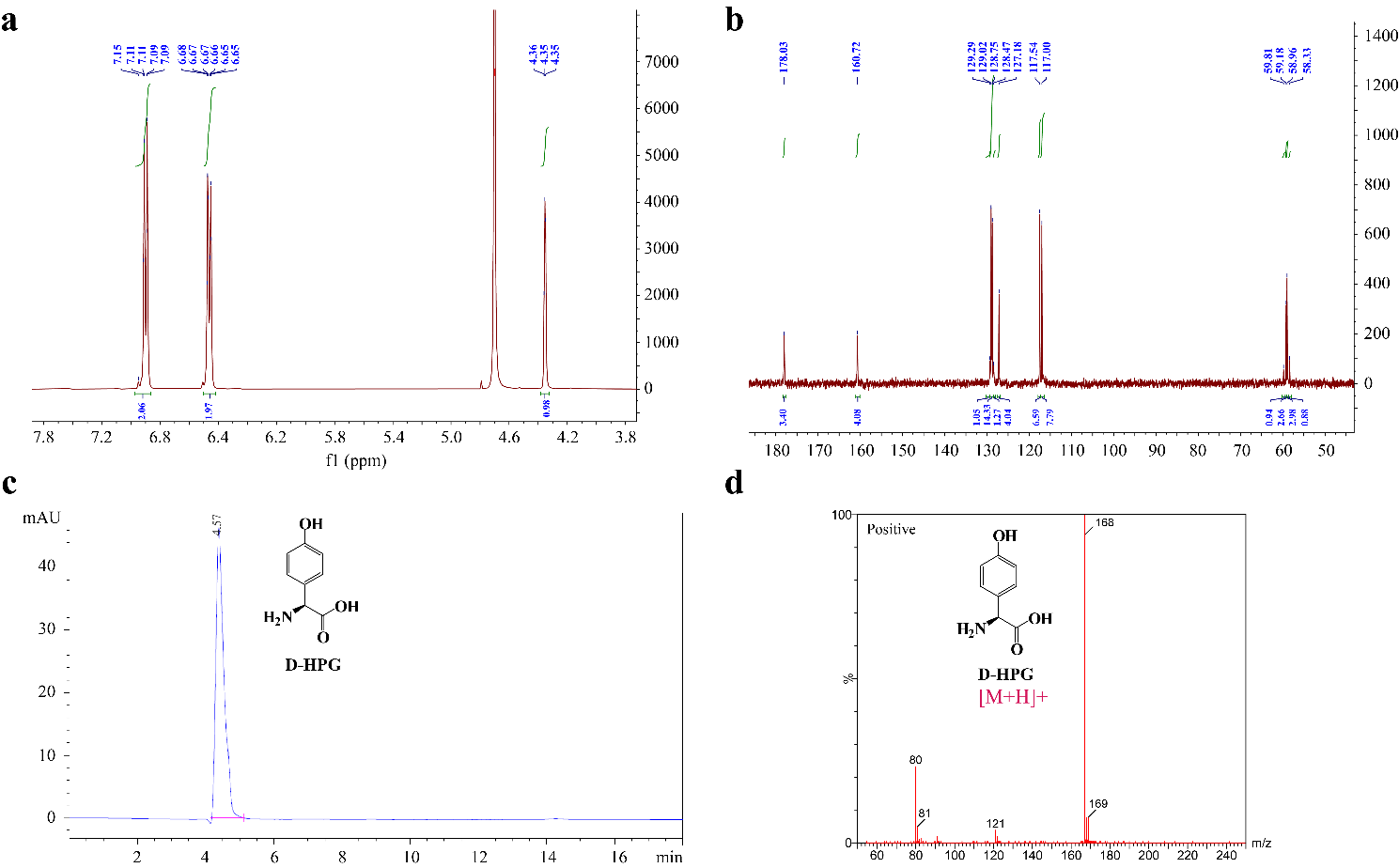


**Fig. S12.** Identity of isolated product. (a) ^1^H-NMR spectra of isolated products. (b) ^13^C-NMR spectra of isolated products. (c) HPLC chromatogram of isolated product. (d) HRMS analysis of isolated product.

# Supplementary materials and methods

## Genetic constructions

All genetic constructions were carried out by using standard molecular biology techniques with PrimeSTAR DNA polymerase, restriction enzymes (*Sac*I, *Sal*I, *Nde*I, *Xho*I, *Hin*dIII, and *Eco*RI) and T4 DNA ligase (all from Takara, China). Heterologous genes were amplified from their respective genomic except for *Pv*L-AAD (GenBank: MK258171), *Pm*L-AAD (GenBank: ADL47009) and *St*DAPDH (GenBank: BAD40410), which were synthesized by GenScript (Piscataway, NJ). The other source strains are listed in **Table S8**. Genomes were extracted from corresponding source strains obtained by culturing first according to the incubation methods of the culture collection centers. The PCR amplification system is showed in **Table S9**. The primers used for gene amplifications are listed in **Table S10**.

## Isolation protocols

The product D-HPG was purified using a Dowex 50WX8 cation exchange column. First, the resin was conditioned by washing with NH_4_OH (2 M, 2×30 mL), HCl (2 M, 2×30 mL) and H_2_O (4×30 mL). Then, the crude reaction mixture was acidified with 1 M HCl and loaded onto the column. Finally, the column was washed with HCl (1M, 2×30 mL), H_2_O (4×30 mL) and eluted with NH_4_OH (2 M, 4×30 mL). Fractions were combined and lyophilized to remove the water, and then separated by preparation thin liquid chromatography (PTLC) with a solvent system of ^n^BuOH/AcOH/H_2_O (4:1:1, by vol.). Silica gel containing D-HPG was collected and eluted with ^n^BuOH/H_2_O (2:1, by vol.). After filtration, the organic solvent was removed by evaporation, and the product was dried overnight under vacuum. The so obtained solid was washed with (EtOH/H_2_O, 9:1, by vol.) affording D-HPG in high chemical purity. The purified products were further identified by NMR, HPLC and HRMS analysis.
